# Self-assembly of N-heterocyclic carbenes on Au(111)

**DOI:** 10.1038/s41467-021-23940-0

**Published:** 2021-06-29

**Authors:** Alex Inayeh, Ryan R. K. Groome, Ishwar Singh, Alex J. Veinot, Felipe Crasto de Lima, Roberto H. Miwa, Cathleen M. Crudden, Alastair B. McLean

**Affiliations:** 1 Department of Physics, Engineering Physics, and Astronomy, Queen’s University, Kingston, ON Canada; 2 Department of Chemistry, Queen’s University, Kingston, ON Canada; 3 Instituto de Física, Universidade Federal de Uberlândia, Uberlândia, Minas Gerais Brazil; 4 Brazilian Nanotechnology National, Laboratory, Campinas, SP Brazil; 5 Institute of Transformative Bio-Molecules (WPI-ITbM), Nagoya University, Chikusa, Nagoya Japan

**Keywords:** Scanning probe microscopy, Surface assembly, Surfaces, interfaces and thin films

## Abstract

Although the self-assembly of organic ligands on gold has been dominated by sulfur-based ligands for decades, a new ligand class, N-heterocyclic carbenes (NHCs), has appeared as an interesting alternative. However, fundamental questions surrounding self-assembly of this new ligand remain unanswered. Herein, we describe the effect of NHC structure, surface coverage, and substrate temperature on mobility, thermal stability, NHC surface geometry, and self-assembly. Analysis of NHC adsorption and self-assembly by scanning tunneling microscopy and density functional theory have revealed the importance of NHC-surface interactions and attractive NHC-NHC interactions on NHC monolayer structures. A remarkable way these interactions manifest is the need for a threshold NHC surface coverage to produce upright, adatom-mediated adsorption motifs with low surface diffusion. NHC wingtip structure is also critical, with primary substituents leading to the formation of flat-lying NHC_2_Au complexes, which have high mobility when isolated, but self-assemble into stable ordered lattices at higher surface concentrations. These and other studies of NHC surface chemistry will be crucial for the success of these next-generation monolayers.

## Abstract (web summary)

Although N-heterocyclic carbenes (NHCs) are a promising class of ligands for forming robust self-assembled monolayers on metals, many questions remain about their behavior on surfaces. Here, the authors address these fundamental questions—such as the factors controlling NHC orientation, mobility, and ability to self-assemble—through an in-depth examination of NHC overlayers on Au(111).

## Introduction

In the almost four decades since the introduction of thiols as ligands for the formation of organic thin films on metals^1^, many of the principles of their self-assembly on gold have been elucidated^2–8^. The same cannot be said for a new type of ligand recently introduced to materials science, N-heterocyclic carbenes (NHCs)^9–21^. NHCs have important advantages compared to thiols, including improved thermal, oxidative, and chemical robustness of their self-assembled monolayers (SAMs); higher binding energies to surfaces; and the ability to bind to reactive metals without ligand decomposition^10–12,14,22^. However, many fundamental properties of NHC-based SAMs are not well-understood, including factors controlling their preferred geometry on gold, their mobility, and their ability to self-assemble.

Several groups have addressed aspects of NHC orientation and organization, pointing to the important role of substituents on the nitrogen atoms adjacent to the carbene carbon (wingtip groups)^11,15,23–25^, however a general picture of what is needed to control orientation is lacking, and the effect of these substituents on self-assembly remains unclear. Moreover, fundamental issues such as whether NHCs bind in an upright fashion to a single gold atom or lie flat on the surface remain contested^12,14,15,19,23–25^. Key issues such as the nature of the bond to the surface, and the mobility of the NHC on the surface have been investigated for specific NHCs^19^, but general principles are missing. Moreover, the influence of the NHC on the underlying gold has not been examined, even though adsorbate-driven destabilization of the herringbone reconstruction^26,27^ is known to be important in generating adatom binding sites for thiol-based SAMs^3,8,13^. These key details are needed for the successful use of NHC-based monolayers in the preparation of dense monolayers, in the production of patterned surfaces, in catalysis, in biosensor preparation, indeed in virtually every application.

**Fig. 1:**
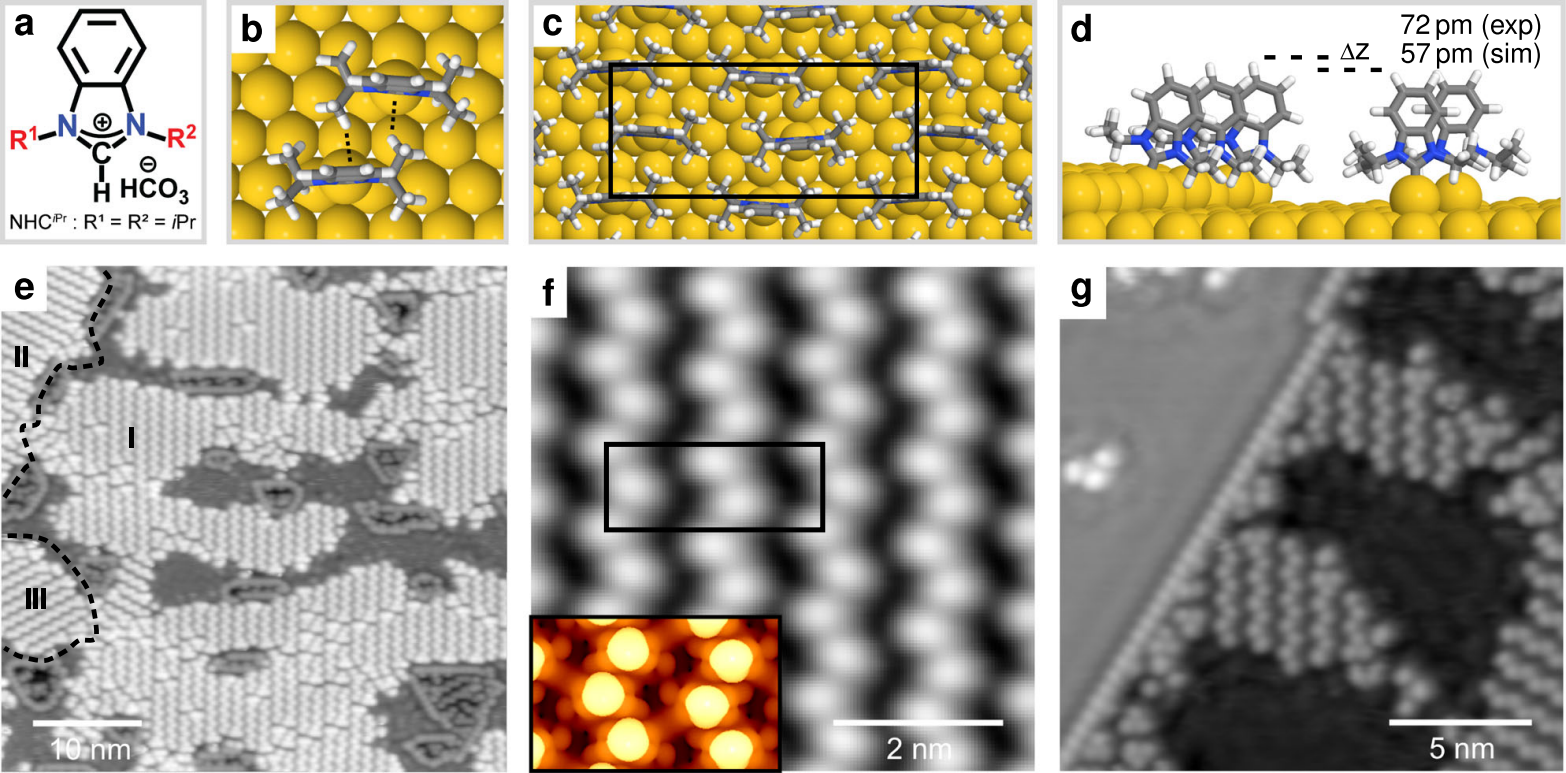
Adatom-mediated self-assembly of NHC^*iPr*^ into zig-zag rows stabilized by non-covalent interactions. **a** Schematic structure of the bicarbonate salt. **b** CH-π interactions between NHC^*iPr*^ molecules on Au(111). **c** Model of the proposed zig-zag lattice. **d** Model showing NHC^*iPr*^ molecules decorating the upper Au(111) step edge together with a zig-zag row on the lower terrace. **e** Scanning tunneling microscropy (STM) image of zig-zag lattice at 0.8 ML coverage with three domains (details: 20 pA tunneling current, 100 mV sample bias, 48.0 × 48.0 nm^2^ scan size, 75 °C anneal temperature). **f** Detail of the zig-zag lattice (80 pA, 50 mV, 6.0 × 6.0 nm^2^, 75 °C). The inset shows an STM simulation of the proposed zig-zag lattice. **g** NHC^*iPr*^ molecules decorating the upper Au(111) step edge together with a zig-zag lattice on the lower terrace (20 pA, 100 mV, 18.0 × 18.0 nm^2^, 125 °C).

To address these issues, we have carried out an in-depth study of 1,3-dialkylbenzimidazol-2-ylidenes, Fig. 1a. NHCs based on this structure have been reported in both flat-lying and upright surface geometries^15,19,23–25^. SAMs of this NHC and closely related structures are known on Au(111)^11,24^, Cu(111)^15^, Ag(111)^14^, and Pt(111)^22^ surfaces, on Au nanoparticles^18,27–32^, in molecular electronics^33–35^, in microcontact printing^36^, and in biosensing applications^12,37^, illustrating the generality and impact of this family of NHCs. Our study illustrates the importance of NHC structure, deposition temperature, annealing temperature, and ligand density, providing much needed information on NHC orientation, mobility, self-assembly, and adatom generation. This study provides information on key factors that influence the self-assembly process, defining conditions needed to prepare high quality, densely packed self-assembled NHC monolayers from this important class of NHC.

## Results

Overlayers of NHC^*iPr*^ were prepared by vacuum deposition of hydrogen carbonate precursors of the general form NHC•H_2_CO_3_, Fig. 1a, onto room temperature Au(111) substrates. The resulting surfaces were imaged by scanning tunneling microscopy (STM) at 77 K, at coverages from sub-monolayer to saturation. Above 0.4 monolayer (ML), NHC^*iPr*^ orders in zig-zag lines on terraces. This lattice, and the NHCs within it, are found to be immobile at 77 K. The study of NHC^*iPr*^ adsorption on Au(111) by density function theory (DFT) (Supplementary Figs. 1–3) identified the energetically preferred binding motif to be an adatom-bound NHC. Structural models based on this binding mode were validated by DFT and used to study the intermolecular forces between molecules in the lattice, Fig. 1b–d. Additionally, surfaces were prepared at saturated coverage for characterization using X-ray photoelectron spectroscopy (XPS) Supplementary section 2.4, Figs. 4–11.

Figure 1e shows a surface with a zig-zag coverage of 0.8 ML, calculated by determining the fraction of available area occupied by upright molecules. On average, the zig-zag lines are 4 nm in length, comprising nine molecules, and span a range of 1 to 12 nm. These zig-zag lines are accompanied by vacancy islands with depth of one atomic step, analogous to those observed in thiol self-assembly^2,7,8^. Inside these islands, NHCs self-assemble into the same zig-zag lattice.

The Au(111) herringbone reconstruction is not clearly identified under the molecular layers, which could indicate that it is significantly distorted or partially lifted by the growth of the zig-zag lattice^26^. In principle, the complete lifting of the herringbone reconstruction can yield up to 0.63 adatoms/nm^2^ ^38^. However, if each NHC^*iPr*^ molecule in the zig-zag lattice is attached to one Au-adatom, a concentration of 1.73 adatoms/nm^2^ is required to form this lattice. Since vacancy islands accompany the zig-zag lattice, these gold atoms likely originate from terraces^2,8^.

Figure 1f is a high-magnification image of the zig-zag lattice, composed of upright NHC^*iPr*^ molecules. In matrix notation, the lattice is best described by (2,2 | –8,8), with the black line in the figure defining a unit cell with dimensions (1.0 ± 0.1) and (2.3 ± 0.1) nm. This lattice possess glide-reflection symmetry, and generates just three domains on an fcc(111) surface (Supplementary Fig. 12). These are equivalent rotational domains, where the long side of the unit cell points along a close-packed $\left\langle {\boldsymbol{1}}\bar{{\boldsymbol{1}}}{\boldsymbol{0}}\right\rangle$ direction, consistent with the domains observed in Fig. 1e.

DFT simulations based upon this lattice, inset to Fig. 1f, are in excellent agreement with experiment. The bright features are found to coincide with the aromatic ring on the NHC^*iPr*^ backbone, with NHC^*iPr*^ attached to Au-adatoms located in threefold hollows, forming zig-zag lines, Fig. 1c, d.

Within each line, both the NHCs and the wingtip groups tilt and rotate into favorable orientations to maximize non-covalent interactions, including: vdW, CH–π^39^, and π–π^40^. The tilting of the NHCs and the rotation of their wingtip groups breaks translational symmetry, doubling the period along the line, and producing unequal zig and zag line segments in the experimental images. The contribution of the zig-zag lattice structure to the difference in line segment length is also captured by DFT (Supplementary Fig. 12).

Figure 1g shows a region containing two terraces separated by a monoatomic step. NHC^*iPr*^ molecules decorate the upper step edge, forming a one-dimensional lattice in which NHC^*iPr*^ binds to every other gold atom. The combination of the unique electronic and steric effects at the step edge enable NHC^*iPr*^ to adopt a purely linear array. Figure 1d shows the calculated conformation of NHC^*iPr*^ in this array, with each NHC exhibiting an ~21° tilt towards the lower terrace, presumably to avoid steric interactions with atoms on the upper terrace.

The proximity of NHC^*iPr*^ molecules on the step edge to those on the surface below enables a direct comparison of their STM heights. A height difference of approximately one atomic step, i.e., 236 pm, would be expected if the NHCs in the zig-zag lattice were attached to surface sites on the lower terrace. However, the observed height difference, measured at a setpoint of 100 mV and 20 pA, is found to be only (72 ± 11) pm. This height difference is corroborated by STM simulations, which take into account the different electronic effects at step edges that may result from variations in the local density of states caused by binding to gold atoms with lower coordination numbers (Supplementary Fig. 13). These results provide conclusive evidence for adatom binding in NHC surface chemistry.

**Fig. 2:**
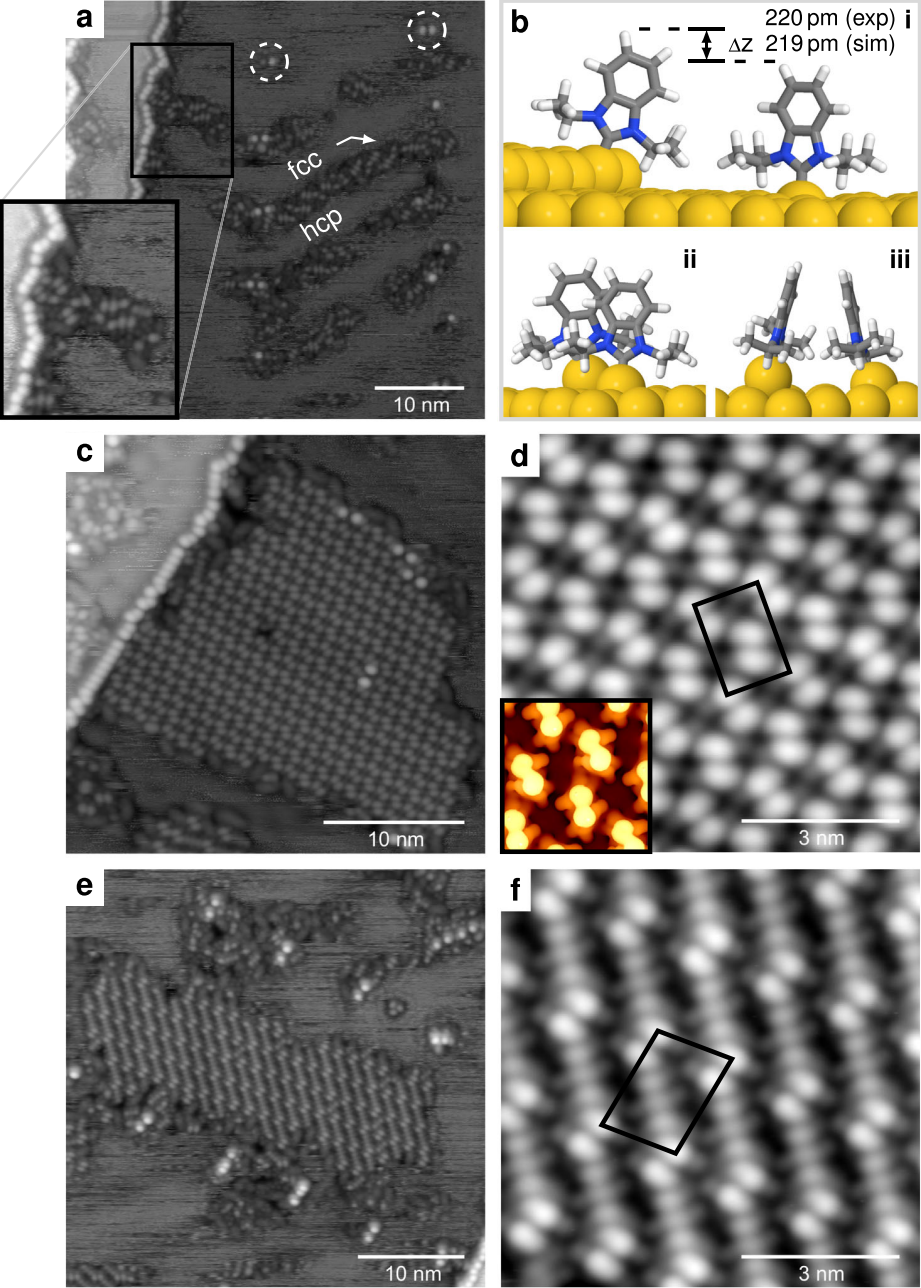
Below the critical coverage required to form zig-zag arrays, NHC^*iPr*^ preferentially binds to surface atoms. **a** In this regime, NHC^*iPr*^ molecules preferentially occupy fcc regions of the herringbone reconstruction (20 pA, 100 mV, 48.0 × 48.0 nm^2^). The inset highlights the STM height difference between NHCs attached to the step and those attached to the lower terrace. **b** Surface binding models showing (i) a surface-bound NHC next to an NHC at the step edge and (ii, iii) NHCs in the proposed surface-bound lattice. The simulated height difference between NHCs at the step edge and NHCs in the surface bound lattice (115 pm) and an isolated, surface-bound NHC (219 pm) are in good agreement with experiment (220 ± 10 pm). **c** Array of surface-bound NHCs on Au(111) (50 pA, 400 mV, 30.0 × 30.0 nm^2^). **d** 8.0 × 8.0 nm^2^ detail. **e** Exotic lattice of mixed surface-bound NHCs and (NHC)_2_Au complexes on Au(111) (20 pA, 100 mV, 40.0 × 40.0 nm^2^). **f** 8.0 × 8.0 nm^2^ detail.

The equilibrium structures self-assembled from NHC^*iPr*^ are critically dependent on coverage. When NHC^*iPr*^ is deposited on Au(111) at room temperature and below ~40% of the saturation coverage, it initially binds to the high-energy adsorption sites found at upper step edges and elbows (Fig. 2a, dotted circles) of the herringbone reconstruction^38^, and then shows a preference for fcc regions^3,8,26^. In this low coverage regime, the zig-zag lattice is not observed, however NHC^*iPr*^ does form other lattices, shown in Fig. 2c–f, with different packing arrangements and adatom involvement.

Comparison of the shape of the features in Fig. 2c with simulations leads to the conclusion that the lattice is composed only of upright NHC^*iPr*^ molecules. Our best estimate of the matrix for the lattice shown is (3,3 | –7,7).

Panel (c) also shows NHC^*iPr*^ molecules decorating the step edge and the elbows of the herringbone reconstruction. A detailed examination of the STM-heights in this image reveals that the NHC^*iPr*^ molecules on the terrace lie (220 ± 10) pm below those bound on the step.

This height difference, comparable to a single step, indicates that in these lattices, NHC^*iPr*^ does not attach to gold adatoms, as it does in the zig-zag lattice, but instead attaches directly to atoms in the surface, lifting them from their equilibrium position. This is illustrated schematically in Fig. 2b where *Δz* represents the origin of the measured height difference. Using the (3,3 | –7,7) matrix extracted from experimental images we performed total energy calculations, and STM simulations based on this lattice are in excellent agreement with experiment and shown in the inset to Fig. 2d.

Figure 2e, f details a more exotic lattice observed at the same low coverage regime. The basis of the unit cell comprises two distinct species that are difficult to identify, however, based on their shape and apparent height we identify them as upright molecules that self-assemble in a dimeric structure, similar to that found in the surface-bound lattice (Fig. 2d), separated by flat-lying complexes with the structure (NHC)_2_Au, where the gold complex center is lifted off the surface. This type of complex has never before been observed for NHC^*iPr*^, although it has precedence in NHCs with smaller substituents^14,15,23,24^.

These three NHC^*iPr*^ lattices (zig-zag, surface-bound, and mixed) differ in structure and in the number of gold atoms incorporated in the overlayer. Estimated Au concentrations for the surface-bound, mixed, and zig-zag lattices are ~0, 0.33, and 1.73 nm^–2^, respectively. Although the zig-zag lattice and the surface-bound lattices are immobile at 77 K, the mixed lattice is not. In STM frames taken 5–10 min apart, structural rearrangement is commonly observed.

To examine how the structure of the wingtip groups influences the self-assembly of NHC^*iPr*^ overlayers, we prepared and deposited structural variants of this NHC in which methyl groups are added (NHC^*t*Bu^), deleted (^Et^NHC^*iPr*^), and in which the isopropyl substituents are exhaustively deuterated (NHC^*iPr*^–*d*_*14*_).

**Fig. 3:**
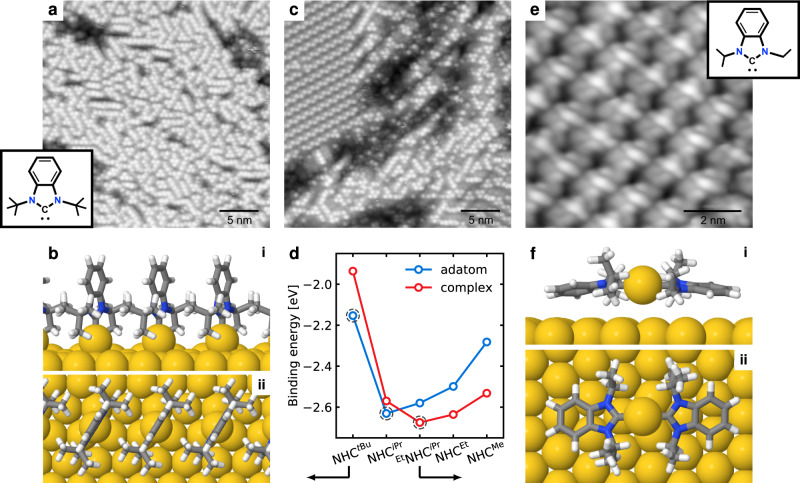
Influence of the wingtip groups on NHC binding and self-assembly. **a** NHC^*tBu*^ self-assembles into short closely packed lines on Au(111) at saturation coverage (20 pA, 100 mV, 30.0 × 30.0 nm^2^). **b** Side and top views of NHC^*tBu*^ in a linear arrangement showing the adatom attachment geometry. **c** Phase-separation of NHC^*iPr*^ and NHC^*tBu*^ molecules co-deposited in equal amounts on Au(111) (20 pA, 600 mV, 30.0 × 30.0 nm^2^). **d** Binding energy per NHC for NHC-adatom and bis-NHC complex configurations for several benzimidazole-based NHCs. **e** ^*Et*^NHC^*iPr*^ forms flat-lying (NHC)_2_Au complexes when deposited onto Au(111) at room temperature (30 pA, 400 mV, 8.0 × 8.0 nm^2^). **f** Side and top views of the (NHC)_2_Au complex centered above a threefold hollow site.

**Fig. 4:**
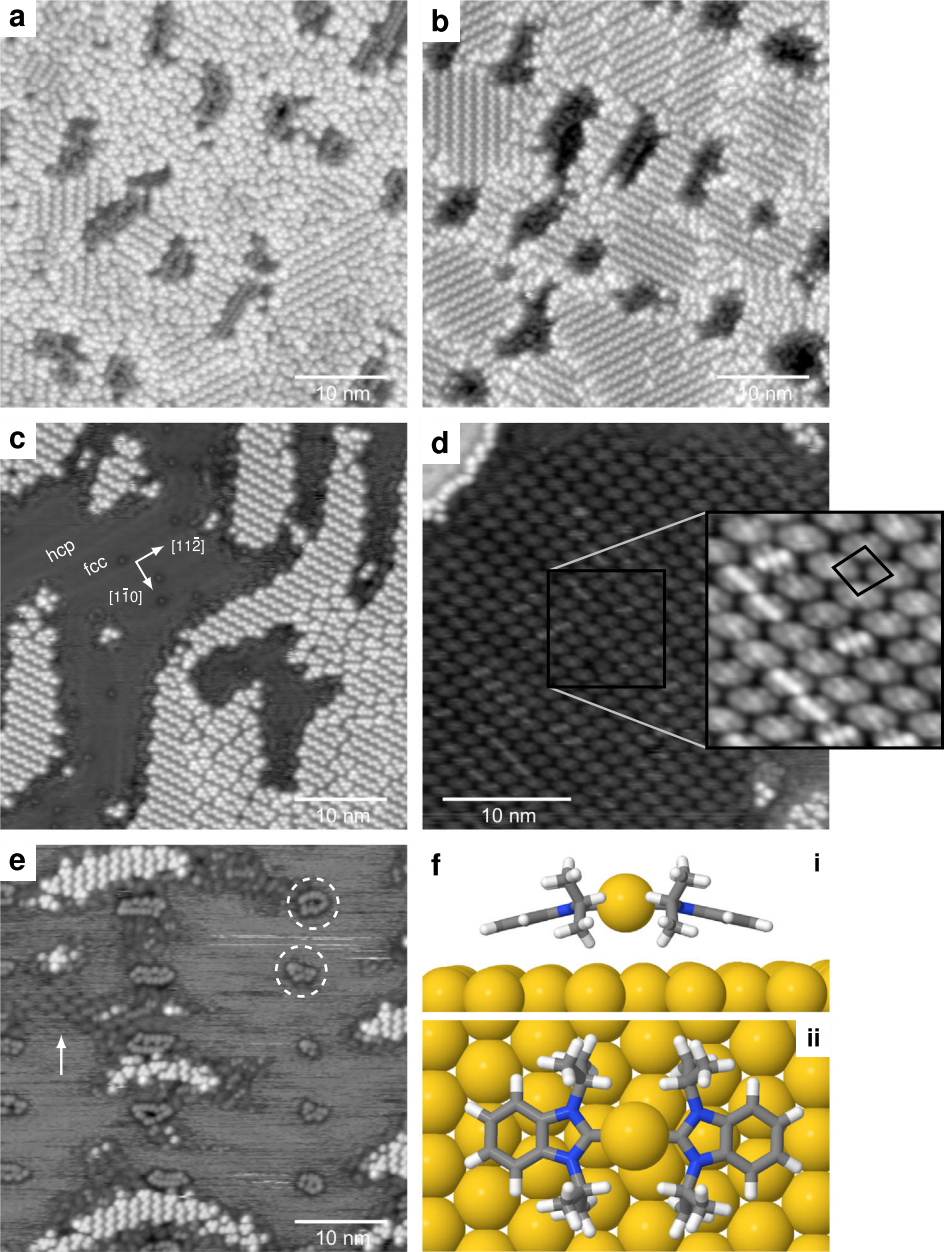
The effect of coverage and temperature on NHC^*iPr*^ adsorption and self-assembly on Au(111). **a** Au(111) surface saturated with upright NHCs (20 pA, 100 mV, 45.0 × 45.0 nm^2^). **b** Subsequent low-temperature annealing produces highly ordered structures (50 °C, 20 pA, 100 mV, 45.0 × 45.0 nm^2^). **c** The coverage of upright species decreases when heating a saturated monolayer to 100 °C (20 pA, 100 mV, 45.0 × 45.0 nm^2^). **d** Large (NHC)_2_Au complex arrays form on Au(111) after heating to high temperature (140 °C, 30 pA, 100 mV, 32.0 × 32.0 nm^2^). **e** (NHC)_2_Au complexes form on room-temperature prepared films at low coverage and self-assemble into small, mobile arrays (20 pA, 100 mV, 45.0 × 45.0 nm^2^). **f** Side and top views of the (NHC)_2_Au complex.

We began with NHC^*t*Bu^, which was deposited onto a room temperature Au(111) surface at saturation coverage, Fig. 3a. In the unannealed state, the NHC^*t*Bu^ overlayer contains similar structural motifs as those formed from NHC^*iPr*^, including trimers, lines, and zig-zag lines (Figs. 3a and 4a). However, the abundance of these structural features is different, with zig-zag lines being more abundant for NHC^*iPr*^ and straight lines for NHC^*t*Bu^. The difference is most easily illustrated using a 50:50 co-deposition on Au(111), Fig. 3c, in which the two NHCs phase-separate.

NHC^*iPr*^ self-assembles into the characteristic zig-zag lattice (Fig. 3c, top left), while NHC^*t*Bu^ forms short lines parallel to the three $\left\langle {\boldsymbol{1}}\bar{{\boldsymbol{1}}}{\boldsymbol{0}}\right\rangle$ directions, comprising, on average, three–five molecules separated by two surface lattice constants, (0.57 ± 0.05) nm. A height difference of 56 ± 9 pm is observed between linear NHC^*t*Bu^ chains and NHC^*iPr*^ overlayers. Such a small difference in height is consistent with both NHC^*iPr*^ and NHC^*t*Bu^ attaching in an upright geometry to adatoms on the surface (Fig. 3d). This value is well-reproduced by DFT for NHC^*iPr*^ in the proposed zig-zag lattice and NHC^*t*Bu^ in a linear arrangement (Supplementary Fig. 13).

Unlike NHC^*iPr*^, which shows a dramatic increase in order upon annealing, NHC^*t*Bu^ cannot be annealed to higher temperatures to increase order, due to appreciable NHC desorption between 50 and 100 °C. Therefore, unlike NHC^*iPr*^, NHC^*t*Bu^ overlayers are far from an equilibrium state, which makes it difficult to know if we have reached the appropriate temperature to enable optimal NHC^*t*Bu^ organization. However, the higher level of organization observed in the NHC^*iPr*^ overlayers may be an inherent property of the smaller *iPr* wingtip groups, which enable the NHCs to sample a larger number of important non-covalent effects such as CH–π interactions, enabling optimization of these interactions.

The NHC^*iPr*^–Au bond is calculated to be more stable than the NHC^*t*Bu^–Au bond by a considerable amount (0.48 eV), which can in large part be attributed to the repulsive steric interactions between NHC^*t*Bu^ and the surface, introduced by the additional methyl groups. The DFT-calculated binding energies shown in Fig. 3d could be used to predict the behavior of all NHCs studied herein, including propensity for adatom binding vs. flat-lying complex formation and ease of thermal desorption, highlighting the power of total energy calculations and the thermodynamic predictability of these systems.

To probe the effect of CH–π interactions on ordering in NHC^*iPr*^ overlayers, we prepared NHC^*iPr*^-*d*_*14*_, in which both isopropyl groups were perdeuterated. Vibrationally averaged CD bonds are only ca. 0.005 Å shorter than CH bonds^41^, and thus any changes observed should be a reflection of alterations to intermolecular hydrogen bonding interactions. However, a unified understanding of the effect of isotopic substitution on CH–π interactions is lacking; with studies of individual molecules suggesting H/D isotope effects are minimal or non-existent^42^. However, a different picture emerges from aggregate and condensed matter systems, which supports diminished strength of interactions for CD–π systems compared with CH–π systems^43^.

Isotopic polymorphism is well known for 3D crystal structures, with pyridine being a common example^44^. In these condensed matter systems, H/D isotopic effects on hydrogen bonding are observed and attributed to a combination of the lower zero-point energy (ZPE) of deuterium compared to protium, and the anharmonicity of the proton/deuteron vibrations. For 3D crystals, intermolecular interactions are often anharmonic resulting in deuterated bridges that are generally weaker than protonated bridges^45,46^. An important exception is when the potential energy surface is harmonic or close to harmonic, in which case deuterated bridges are stronger. Since a self-assembled monolayer of NHCs can be considered as a 2D crystal, similar rules are expected to apply, and for the CH–π interactions examined, the proton/deutron motion is anharmonic, therefore deuterated bridges should be weaker in our assembly.

Indeed when NHC^*iPr*^–*d*_*14*_ was deposited on Au(111), an overlayer without any long range ordering was observed (Supplementary Fig. 14). Thus, these results may be interpreted as supporting the importance of CH–π effects on the self-assembly of NHC^*iPr*^.

Structural variant ^Et^NHC^*iPr*^ was examined next. This NHC is related to NHC^*iPr*^, but with a single methyl deletion. When deposited onto a room temperature surface, ^Et^NHC^*iPr*^ produces exclusively flat-lying (NHC)_2_Au complexes with no upright NHCs observed, Fig. 3e, a remarkable result considering the triviality of the structural difference. DFT comparisons of upright binding modes versus flat-lying complexes indicate that for ^Et^NHC^*iPr*^, bis-NHC complexes should be more stable than upright species, Fig. 3d. These calculations also point to significant thermodynamic preferences for bis-NHC complexes with NHCs bearing methyl and ethyl wingtip groups, consistent with literature reports^14,15,23,24^, and preference for the upright geometry with NHC^*t*Bu^. Interestingly, among all NHCs examined, NHC^*iPr*^ has the smallest energetic difference between the upright and flat-lying species, Fig. 3d, suggesting that both should be energetically accessible.

Overlayers composed of NHC^*iPr*^, NHC^*t*Bu^, and ^Et^NHC^*iPr*^ were also examined by XPS to obtain spectroscopic support for the STM results presented herein. As shown in Supplementary Information section 2.4, a significant difference was observed between NHCs known to form upright overlayers and those giving flat-lying NHC_2_Au complexes. Specifically, NHC^*iPr*^ and NHC^*t*Bu^ overlayers gave film thicknesses of 0.90 ± 0.08 and 0.85 ± 0.03 nm, respectively, which corresponds well with what is expected for an upright geometry, while films of ^Et^NHC^*iPr*^ had a measured thickness of 0.17 ± 0.11 nm. These results are consistent with the observations made by STM.

To test for the possible interconversion of the upright and flat-lying species, overlayers of NHC^*iPr*^ were heated above room temperature. Although NHC^*iPr*^ self-assembles into zig-zag arrays when deposited onto Au(111) at room temperature, further annealing to 50 °C promotes an increase in order, Fig. 4a, b. Remarkably, heating to only 75 °C is sufficient to convert some NHCs in the zig-zag lattice into (NHC)_2_Au complexes. This process occurs irreversibly, with the number of complexes observed at 77 K increasing with the anneal temperature. As shown in Fig. 4c, heating also reduces both the number and the size of the vacancy islands. This surface was heated to 100 °C and contains evidence for (NHC)_2_Au complexes that were captured in motion around the perimeter of the zig-zag lattice. Flat-lying (NHC)_2_Au complexes have never previously been observed by STM for NHCs bearing anything but primary substituents (Me, Et, or *n*-Bu)^14,15,23,24^, and a reduction-triggered transition from upright to flat-lying has been proposed for a nitroarene–substituted NHC^47^.

At 77 K, unlike the zig-zag lattice, the complex lattice (Fig. 4d) is only resolvable when the complexes are spatially constrained, e.g., when the excluded surface area becomes vanishingly small, suggesting that (NHC)_2_Au complexes are mobile at this temperature. Several candidates for the experimentally observed lattice have been identified (Supplementary Table 4, Fig. 15). We also observe the rotation of complete domains (Supplementary Fig. 16) suggesting that, at 77 K, the activation energy barriers for both complex translation and rotation are not large enough to freeze out translational and rotational changes of state. The observed change in conformational state is in sharp contrast to the upright surface bound-and adatom-bound lattices, which were found to be stable for days and resisted any attempts at manipulation with the STM tip, which included changing the set current and voltage and the scan speed and direction.

STM images prepared at low NHC^*iPr*^ coverage on room temperature surfaces also contain (NHC)_2_Au complexes, Fig. 4e, suggesting that these species are more ubiquitous than previously thought. In fact, only surfaces that have a saturation coverage of the zig-zag lattice are devoid of complexes, Fig. 4a. Since complexes are mobile, they clearly play an important role in surface mass transport (Supplementary Fig. 17).

## Discussion

This in-depth examination of NHC self-assembly on Au(111) enables a number of conclusions about the behavior of NHCs on Au(111) surfaces, the stability and mobility of NHC overlayers, and the factors that control NHC speciation and surface organization.

At room temperature, and below a critical coverage, NHC^*iPr*^ binds primarily to surface sites, lifting surface atoms from their equilibrium positions by as much as 167 pm^11,19,28^. Above 0.4 ML, NHC^*iPr*^ self-assembles into trimers, lines, and a zig-zag lattice comprised of NHCs that bind exclusively to adatoms, the thermodynamically predicted attachment geometry. The abundance of the zig-zag lattice relative to other surface motifs can be increased by annealing to temperatures as low as 50 °C. This lattice is found to be stable and immobile at 77 K. The finding that a critical coverage is needed to generate the lattice is presumably related to the distortion or partial lifting of the herringbone by the well-known process of adsorbate-induced stress modification^3,8,26,27,47^. The ability of NHCs to bind to surface atoms or via adatoms is conclusively illustrated by measurements of the STM heights of NHCs on step edges as compared to those on terraces.

Both NHC^*iPr*^ and NHC^*t*Bu^ bind to the surface in an upright fashion forming isomorphic structural motifs such as trimers, lines, and zig-zag rows. The effect of adding an additional methyl group is dramatic. It significantly decreases the binding energy of the NHC to the surface, preventing the system from achieving equilibrium, and restricts intramolecular rotations, complicating the ability to achieve long range order.

DFT calculations highlight the importance of CH–π interactions between the wingtip substituents and adjacent benzimidazolylidene rings and π–π interactions between the benzimidazolylidene rings. The impact of hydrogen bonding in NHC^*iPr*^ overlayers is clearly illustrated by deposition of the perdeuterated analog NHC^*iPr*^–*d*_*14*_, which showed upright bonding but no long-range order.

Less sterically constrained NHCs, such as ^Et^NHC^*iPr*^, show the exclusive formation of flat-lying (NHC)_2_Au complexes that are mobile at 77 K. DFT calculations show that for ^Et^NHC^*iPr*^, and all other NHCs with less bulky wingtip groups, the flat-lying complex is thermodynamically preferred. This finding is of significant importance since NHCs are largely assumed to bind in an upright manner regardless of wingtip structure and the behavior of NHCs in these two binding modes are shown to be very different.

Among those NHCs studied, NHC^*iPr*^ occupies a unique position, with both upright and flat-lying geometries predicted to be energetically accessible. Consequently, we observe a transition from a stable, upright, zig-zag lattice to a flat-lying (NHC)_2_Au complex upon annealing.

Through this study, we have demonstrated that the formation of high quality, densely packed NHC SAMs can be achieved after careful consideration of both covalent and non-covalent interactions. Differences in wingtip structure determine binding geometry, mobility, overlayer ordering, and the adatom extraction phenomenon. In particular, wingtip groups must be large enough to disfavor flat–lying complexes, but not so large that they weaken the bond to the surface. The ability to form inter-NHC interactions and to withstand annealing is important for the formation of highly ordered, upright lattices, and the specific effect of CH–π interactions have been highlighted. These details advance the understanding of NHC-based SAMs and will be crucial to predict and control NHC SAM properties in future applications.

## Methods

### Syntheses

Reactions were performed using reagent grade solvents, with exceptions of methanol where HPLC-grade was used. Work-up purifications were performed using commercial reagent-grade solvents. Benzimidazole, chloroform-*d*, and methanol-*d*_*4*_ were purchased from Sigma-Aldrich and used without any purification. High-resolution mass spectrometric (HRMS) data were obtained from a Thermo Fischer Scientific Exactive (ESI) or Applied Biosystems/MDX ScieX QSTar QqTOF instrument (EI). ^1^H and ^13^C NMR spectra were recorded on Bruker Instruments operating at denoted spectrometer frequency given in megahertz (MHz) at 25 °C. ^1^H NMR chemical shifts are referenced to the residual protons of the deuterated solvents CDCl_3_ (at *δ* = 7.26 ppm) and CD_3_OD (at *δ* = 3.31 ppm); ^13^C chemical shifts are referenced to the CDCl_3_ and CD_3_OD signals at *δ* = 77.16 and 49.00 ppm, respectively. IR spectra were collected on an Agilent Cary 630 FTIR as solids and absorption bands are given in cm^–1^. Elemental analyses were performed using Flash 2000 CHNS-O analyzer.

Amberlyst A26 hydroxide resin was obtained from Sigma-Aldrich and converted to hydrogen carbonate anion exchange resin (Resin-HCO_3_) following a previously reported procedure^12^. Using this anion exchange resin, the benzimidazolium hydrogen carbonate salts NHC^*iPr*^^12^, was prepared from its corresponding iodide salt following previously reported literature procedures^12,48^.

NHC^*iPr*^–*d*_*14*_ was prepared from the corresponding bromide salt^49^, however the anion exchange was performed using our standard resin protocol (see Supplementary Information for details). NHC^tBu^ was prepared from the corresponding chloride salt,^50^ however the anion exchange was performed using our standard resin protocol (see Supplementary Information for details).

### XPS measurements

XPS spectra were recorded on a Kratos Nova AXIS spectrometer equipped with AlN X-ray source. Samples were mounted on an aluminum sample holder using double-sided adhesive copper tape and kept under high vacuum (10^–9^ Torr) overnight inside the preparation chamber before being transferred to the analysis chamber (ultra-high vacuum, 10^–10^ Torr). Data were collected using Al K_α_ radiation operating at 1486.69 eV (150 W, 15 kV), charge neutralizer and a delay-line detector (DLD) consisting of three multichannel plates. Acquired data was processed using CasaXPS software following reference handbooks^51,52^. The thickness of SAMs containing NHCs with various substituents was measured using method described by Bain and Nuzzo^53,54^. We assumed that NHC-monolayers would attenuate the Au4f signal like thiol-based monolayers. The thickness of SAMs was found to be representative to the molecular geometry on surface. These data are reported in Supplementary Information. Trends from these data suggest that there are differences between monolayers formed from NHCs with different wingtip groups. The authors would like to point that absolute values of thickness measurements were not confirmed by ellipsometry or ARXPS, therefore, they should be taken as tentative only.

### Computational approach

The calculations of atomic geometry were performed based on the DFT approach, as implemented in the VASP code^55^. The exchange correlation term was described using the GGA functional proposed by Perdew, Burke, and Ernzerhof^56^. The Kohn–Sham orbitals are expanded in a plane wave basis set with an energy cutoff of 400 eV. The Brillouin zone is sampled according to the Monkhorst–Pack method^57^, using a gamma-centered 3 × 3 x 1 mesh. The electron-ion interactions are taken into account using the Projector Augmented Wave method^58^. We have considered a Au(111) slab with four atomic layers of which the two lower layers were fixed in the bulk lattice parameter, while the others are allowed to relax. All NHC/Au geometries have been relaxed until the atomic forces on each atom were less than 0.02 eV/Å. In order to take into account the dispersion forces, we have considered non-local van der Waals interactions included throughout vdW-DF functional^59^. For the simulations of the upstanding molecules isolated in the Au(111) surface we have used a supercell with 5 × 5 periodicity, wielding a 14.7 Å distance between its periodic images and a vacuum region of 15 Å, while for the bis-NHC complex we have considered a 7 × 7 surface periodicity, resulting in a lateral distance of 20.6 Å between the molecule and the periodic images, and a vacuum region of 18 Å. Such supercells have shown to be sufficient to avoid spurious interactions; resulting in a convergence of less than 3 meV on the absolute values of the adsorption energies when compared with larger supercells.

### STM measurements

STM measurements were performed using (i) a CreaTec low-temperature scanning probe microscope (LT-SPM), (ii) a SPECS Nanonis BP5 Controller, and (iii) a FEMTO DLPCA-200 preamplifier. STM scans were performed in UHV at 77 K using a bath cryostat filled with liquid nitrogen. A mechanically cut Pt(90%)Ir(10%) tip was used, sharpened in situ via plunges into the gold crystal. Constant-current feedback mode was used with the following typical set of operating conditions: a set current of 20 pA; a bias voltage of 100 mV (sample voltage with respect to the tip); and a scan speed of 100–300 nm/s. A real-time method for drift compensation was implemented based on the TurboReg/StackReg plugin^60^ that calculated the drift vector during scanning by registering (aligning) multichannel STM images. Subsequent image processing was performed without the use of filters (e.g., FFT filtering), and included further drift correction if needed and Gaussian blurring. Height value and x and y center value distributions were determined from image thresholding analysis based on height parametrization, from which statistical quantities including lattice constants were determined.

## Supplementary information

Supplementary Information

## Data Availability

The authors declare that all the important data to support the findings in this paper are available within the main text or in the supplementary information. Extra data are available from the corresponding author upon reasonable request.
