# Supplementary material for: Self-assembly of N-heterocyclic carbenes on Au(111)
Source: Nat Commun. 2021 Jun 29;12:4034. doi: 10.1038/s41467-021-23940-0 (PMC8241988; doi:10.1038/s41467-021-23940-0)
Supplement: Supplementary file 1 — Supplementary Information [file 41467_2021_23940_MOESM1_ESM.pdf]

Self-assembly of *N*-heterocyclic carbenes on Au(111)

Supplemental information

Alex Inayeh[1], Ryan R. K. Groome[1], Ishwar Singh[2], Alex J. Veinot[2], Felipe Crasto de Lima[3], Roberto H. Miwa[3] Cathleen M. Crudden[2] & Alastair B. McLean[1]

[1]Department of Physics, Engineering Physics and Astronomy, Queen's University, Stirling Hall, Kingston, Ontario, K7L 3N6, Canada.

[2]Department of Chemistry, Queen's University, Chernoff Hall, 90 Bader Lane, Kingston, Ontario K7L 3N6, Canada.

[3]Instituto de Física, Universidade Federal de Uberlândia, CP 593, 38400-902, Uberlândia, MG, Brazil.

May 2, 2021

Contents

Contents **2**

1 Supplementary methods **3**

1.1 Synthesis 3

1.2 Sample preparation 6

2 Figures **8**

2.1 Overview of NHC^{iPr} binding and self-assembly 8

2.2 Calculation of binding energy 10

2.3 Calculation of charge density 12

2.4 X-ray photoelectron spectroscopy 13

2.5 The NHC^{iPr} zig-zag lattice 22

2.6 Height difference calculations 24

2.7 Fine-tuning the intermolecular interactions of NHC^{iPr} 27

2.8 Complex self-assembly configurations 29

2.9 Complex lattice instabilities 31

2.10 Diffusion of upright NHC^{iPr} and bis-NHC^{iPr} complexes 33

Supplementary references **35**

1 Supplementary methods

1.1 Synthesis

Preparation of 1,3-di-(propan-2-yl-d_7)-benzimidazolium hydrogen carbonate

1,3-di-(propan-2-yl-d_7)-benzimidazolium hydrogen carbonate (NHC^{iPr}-d_{14}) was prepared following a modified literature procedure [1]. 1,3-di-(propan-2-yl-d_7)-benzimidazolium bromide (74 mg, 0.25 mmol, 1 eq.) was dissolved in 3 mL of methanol, producing a yellow coloured solution. Freshly activated resin-HCO_3 (2 mL, 6 eq.) was washed with methanol (3×2 mL). The yellow coloured solution of bromide salt was added to the activated resin and this mixture was left to stir at room temperature for 30 minutes. The beads were then removed by filtration and washed with methanol (4×5 mL) before evaporating the solvent using a stream of compressed air overnight. The resultant yellow oil was then triturated with acetone (3×4 mL) and dried *in vacuo* to afford the desired product as a beige powder (52 mg, 74 %).

^{1}H NMR (700 MHz, CD_3OD) δ: 8.05-8.03 (m, 2H, aromatic C-H), 7.73-7.71 (m, 2H, aromatic C-H). The C(2) position could not be detected presumably due to exchange with the solvent.

^{13}C NMR (176 MHz, CD_3OD) δ: 162.61, 139.16, 132.62, 128.17, 114.97, 57.48 (*t*, $^1J_{CD}$ = 21.6 Hz), 17.27 (*p*-observed, $^1J_{CD}$ = 19.4 Hz).

FTIR: Strong peaks for CO_2 asym. str. at 1617.30 cm^{-1} and sym. str. at 1371.00 cm^{-1}. C-D str. at 2251 cm^{-1}.

Spectroscopic data were consistent with published report [2].

Preparation of 1,3-di-tert-butylbenzimidazolium hydrogen carbonate

1,3-Ditertbutylbenzimidazolium hydrogen carbonate (NHC^{tBu}) was prepared following a modified literature procedure [1]. 1,3-Di-tert-butylbenzimidazolium chloride (70 mg, 0.26 mmol, 1 eq.) was dissolved in 5 mL of methanol, producing a yellow coloured solution. Freshly activated resin-HCO_3 (1 mL, 3 eq.) was washed with methanol (3×2 mL). The yellow coloured solution of chloride salt was added to the activated resin and this mixture was

left to stir at room temperature for 30 minutes. The beads were then removed by filtration and washed with methanol (3×5 mL) before evaporating the solvent using a stream of compressed air. The resultant yellow coloured solids were then triturated with ether (3×10 mL) and dried *in vacuo* to afford the desired product as a colourless powder (40 mg, 53 %).

^{1}H NMR (400 MHz, CD_3OD) δ: 8.29–8.24 (m, 2H, aromatic C-H), 7.72–7.67 (m, 2H, aromatic C-H), 1.91 ppm (s, 18H, $C(CH_3)_3$). The C(2) position could not be detected presumably due to exchange with the solvent.

^{13}C{^{1}H} NMR (100 MHz, CD_3OD) δ: 132.9, 127.4, 117.8, 62.8, 28.9 ppm. The C(2) position and the bicarbonate anion could not be detected presumably due to exchange with the solvent.

FTIR: Strong peaks for CO_2 asym. str. at 1613.51 cm^{-1} and sym. str. at 1371.30 cm^{-1}.

HRMS (m/z) for $C_{15}H_{23}N_2^+$ $[M\text{-}HCO_3]^+$: 231.1851, calc.: 231.1855.

Anal. Calc. for $C_{13}H_{18}N_2O_3$: C, 65.73 %; H, 8.27 %; N, 9.58 %. Found: C, 65.80 %; H, 8.19 %; N, 9.68 %.

Preparation of 1-ethyl-3-isopropylbenzimidazolium iodide

1-ethyl-3-isopropylbenzimidazolium iodide was prepared by combining 1-isopropylbenzimidazole (2.34 g, 14.6 mmol, 1 eq.) and iodoethane (3.5 mL, 43.8 mmol, 3 eq.) in a 15 mL PTFE-capped pressure tube. The resultant yellow coloured mixture was left to stir at 100 °C for 18 hours. After removal of the excess iodoethane *in vacuo*, the orange coloured solids were collected and triturated with hexanes (4×10 mL) to afford a colourless powder (3.14 g, 68 %).

^{1}H NMR (400 MHz, $CDCl_3$) δ: 10.82 (s, 1H, N_2CH), 7.85–7.76 (m, 2H, aromatic C-H), 7.65–7.60 (m, 2H, aromatic C-H), 5.06 (sept, 1H, $^3J_{HH}$ = 6.7 Hz, CH-$(CH_3)_2$), 4.61–4.55 (q, 2H, $^3J_{HH}$ = 7.4 Hz, CH_2CH_3), 1.73 (d, 6H, $^3J_{HH}$ = 6.7 Hz, CH-$(CH_3)_2$), 1.66 ppm (t, 3H, $^3J_{HH}$ = 7.4 Hz, CH_2CH_3).

$^{13}C\{^1H\}$ NMR (100 MHz, $CDCl_3$) δ: 140.0, 131.3, 130.6, 127.3, 127.2, 113.8, 113.3, 51.9, 41.2, 22.4, 15.1 ppm.

HRMS (m/z) for $C_{12}H_{17}N_2^+$ $[M\text{-}I]^+$: 189.1314, calc.: 189.1387.

Anal. Calc. for $C_{12}H_{17}N_2I$: C, 45.58 %; H, 5.42 %; N, 8.86 %. Found: C, 46.20 %; H, 5.42 %; N, 8.68 %.

Preparation of 1-ethyl-3-isopropylbenzimidazolium hydrogen carbonate

$^{Et}NHC^{iPr}$ was prepared following a modified literature procedure [1]. 1-ethyl-3-isopropylbenzimidazolium iodide (320 mg, 1.0 mmol, 1 eq.) was dissolved in 5 mL of methanol, producing a yellow coloured solution. Freshly activated resin-HCO_3 (4 mL, 3 eq.) was washed with methanol (3 × 5 mL). The yellow coloured solution of iodide salt was added to the activated resin and this mixture was left to stir at room temperature for 30 minutes. The beads were then removed by filtration and washed with methanol (3 × 5 mL) before evaporating the solvent using a stream of compressed air. The resultant yellow coloured solids were then triturated with acetone (3 × 10 mL) and dried *in vacuo* to afford the desired product as a colourless powder (150 mg, 60 %).

^1H NMR (400 MHz, CD_3OD) δ: 8.06–7.99 (m, 2H, aromatic C-H), 7.75–7.71 (m, 2H, aromatic C-H), 5.07 (sept, 1H, $^3J_{HH}$ = 6.7 Hz, CH-$(CH_3)_2$), 4.61–4.55 (m, overlapping quartets, 2H, $^3J_{HH}$ = 7.4 Hz, CH_2CH_3), 1.73 (d, 6H, $^3J_{HH}$ = 6.7 Hz, CH-$(CH_3)_2$), 1.66 ppm (t, 3H, $^3J_{HH}$ = 7.4 Hz, CH_2CH_3). The C(2) position could not be detected presumably due to exchange with the solvent.

$^{13}C\{^1H\}$ NMR (100 MHz, CD_3OD) δ: 161.4, 133.0, 132.5, 128.2, 128.1, 114.9, 114.5, 52.6, 43.8, 22.1, 14.8 ppm. The C(2) position could not be detected presumably due to exchange with the solvent.

FTIR: Strong peaks for CO_2 asym. str. at 1624.69 cm^{-1} and sym. str. at 1367.57 cm^{-1}.

HRMS (m/z) for $C_{12}H_{17}N_2^+$ $[M\text{-}HCO_3]^+$: 189.13814, calc.: 189.1387.

Anal. Calc. for $C_{13}H_{18}N_2O_3$: C, 62.38 %; H, 7.25 %; N, 11.19 %. Found: C, 60.94 %; H, 7.42 %; N, 11.20 %.

1.2 Sample preparation

X-ray photoelectron spectroscopy

Prior to SAM deposition, Au-substrates (Au/Ti/Si) were electrochemically cleaned to remove any adsorbed surface contaminants. Base cleaning was performed by dipping Au-substrate in 0.5 M KOH (aq) and by cycling current between 0 and -2 V at a scan rate of 0.5 V^{-1} for 100 cycles, followed by 100 cycles in 0.5 M H_2SO_4 (aq) between 0 and 1.5 V. Au-substrates were then thoroughly rinsed with Milli-Q water to remove any trace acid and HPLC-grade MeOH to remove excess water. Substrates were then dried using a stream of dry Argon (Praxair, 5.0).

Additionally, we prepared and compared SAMs on Au/Mica to observe any differences due to change of underlying substrate. We obtained indistinguishable results from both substrates, therefore, we continued using Au/Ti/Si, because they were easier to handle.

SAMs were prepared by flash vacuum deposition of **NHC•H_2CO_3** by placing Au-substrate in line-of-sight and heating the powder under a dynamic vacuum. Binding energy shifts and atomic percentages for O 1s, N 1s, C 1s and Au 4f regions are in Supplementary Table 1 and 2 respectively.

Scanning tunneling microscopy

NHC•H_2CO_3 was added to ethanol and one droplet of this solution was placed at the center of an oxidised silicon wafer. The NHCs were flash evaporated in high-vacuum (10^{-7} mbar) from the silicon wafer onto clean Au(111) held at room temperature, 135 K, or 5 K, by passing a high current (3.0 A) through the wafer for 5 s. The surface coverage was controlled by varying the molarity of the solution between 5.0 and 42.5 mM.

The Au(111) surface, a MaTecK gold single crystal, was cleaned and atomically ordered through repeated cycles of Ar ion sputtering (1.5 keV for 5 minutes) and radiative heating to 575°C at a rate of 100°C/min over 5 mins. The ramp-rate was reduced to (10 ± 2)°C/min when heating the sample post-deposition. Once the sample reached the target temperature, the heater was turned off and the sample was transferred to the microscope to cool to 77 K.

2 Figures

2.1 Overview of NHC$^{i\mathrm{Pr}}$ binding and self-assembly

This section provides an overview of NHC$^{i\mathrm{Pr}}$ binding and self-assembly behaviour, Supplementary Figure 1. NHC$^{i\mathrm{Pr}}$ shows a preference for step edges and elbow sites of the herringbone reconstruction at low coverages. STM image sequences show that the molecules found at these adsorption sites, which are the most reactive sites on Au(111), are mostly immobile. STM observations also indicate the presence of upright, surface-bound NHCs, flat-lying $(NHC)_2Au$ complexes, and NHC monomers on terraces at low coverages. These species form a highly mobile 2D gas-like phase before self-assembling into lattices that are stable for imaging. By increasing the coverage slightly, we observed lattices composed of surface-bound NHCs, $(NHC)_2Au$ complexes, and a more exotic lattice possibly involving both surface-bound NHCs and $(NHC)_2Au$ complexes.

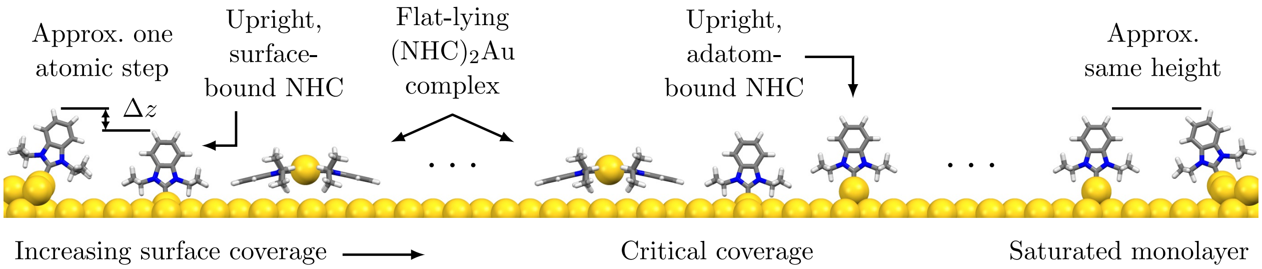

Supplementary Figure 1: Structural model showing the different adsorption configurations of NHC$^{i\mathrm{Pr}}$ on Au(111) as a function of surface coverage.

Further increase in surface coverage led to the formation of the zig-zag lattice, consisting of upright, adatom-bound NHCs. A number of observations support adatom involvement, including: (1) the observation of vacancy islands, which form by the removal of Au atoms from the top surface layer, (2) the structure of the zig-zag lattice, which suggests NHCs sit above three-fold hollow sites, (3) the small apparent height difference between NHCs at step edges and NHCs in the zig-zag lattice, and (4) agreement with DFT calculations. Together, our combined STM/DFT study provides a consistent picture for NHC attachment to gold

adatoms.

The critical coverage needed to generate the zig-zag lattice is found to be approximately 0.4 ML. At this coverage, the STM data also show evidence for surface-bound NHCs and small arrays of $(NHC)_2Au$ complexes. We found that their relative abundance on the surface is considerably affected by coverage, possibly due to competitive adsorption or on-surface chemical reactions. On saturated surfaces, we observe a single phase consisting of upright, adatom-bound NHCs. The thermal treatment of NHC-covered films showed significant increase in ordering upon heating to 50°C. With further annealing, vacancy islands progressively disappeared and $(NHC)_2Au$ complexes formed at the expense of the zig-zag lattice.

2.2 Calculation of binding energy

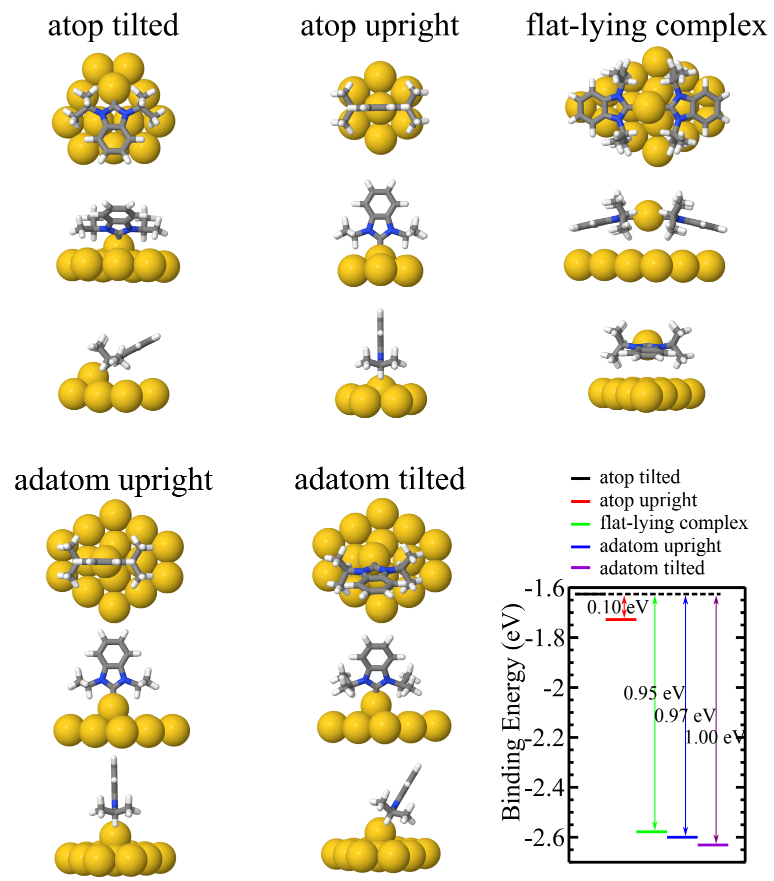

Supplementary Figure 2: Atomic geometries and binding energies for NHC$^{i\text{Pr}}$ on Au(111). For clarity, only part of the (111) surface has been drawn for each geometry. The calculated binding energies have been plotted in the graph located bottom right. The adatom tilted geometry is the most stable and the atop tilted (i.e. bound to an atom in the surface layer) is the least stable attachment geometry. However, the binding energy of the adatom upright and the flat-lying complex attachment geometries are comparable.

In order to understand the NHC$^{i\text{Pr}}$/Au(111) molecule/surface interaction, we performed *first-principles* simulations using density functional theory. Details of the calculation approach are presented above. We have examined five NHC$^{i\text{Pr}}$/Au(111) configurations for isolated molecules or bis-NHC$^{i\text{Pr}}$ complexes, shown in Supplementary Figure 2, labeled as: atop tilted, atop upright, flat-lying complex, adatom upright, and adatom tilted. The strength of the molecule–surface interaction was inferred through the calculation of the binding energy

(E^b) defined as,

$$E^b = E_{\mathrm{iPr/Au}} - E_{\mathrm{iPr}} - E_{\mathrm{Au}}, \tag{1}$$

which is a total energy comparison between the final system ($E_{\mathrm{iPr/Au}}$), and the separated components, NHC$^{i\mathrm{Pr}}$ molecule or complex (E_{iPr}), and Au(111) pristine surface or upon the presence of an adatom (E_{Au}). Our results for the binding energy, summarized in the lower right panel of Supplementary Figure 2, reveal a preference for the adatom tilted configuration ($E^b = -2.63\,\mathrm{eV/NHC}^{i\mathrm{Pr}}$), followed by the adatom upright ($E^b = -2.60\,\mathrm{eV/NHC}^{i\mathrm{Pr}}$). In the conformations we have considered, the adatom is adsorbed in the fcc hollow site; in the hcp hollow site the binding energy is 14 meV weaker. The flat-lying complex ($E^b = -2.58\,\mathrm{eV/NHC}^{i\mathrm{Pr}}$) is predicted to be a likely configuration. Those structures are somewhat close in energy, being the former (latter) most (least) stable by ∼50 meV/NHC. The NHC$^{i\mathrm{Pr}}$ molecules bonded directly to the bare Au(111) surface, *i.e.* atop tilted and atop upright, present less stable binding interaction due steric repulsion between the NHC$^{i\mathrm{Pr}}$ wingtip group and the Au surface. For instance, in the atop upright configuration, the Au atom is pulled out by ∼1 Å from the surface layer, resulting in a larger C–Au bond length (2.15 Å) compared with that of the adatom upright geometry, 2.06 Å. In order to provide a figure-of-merit for steric repulsion, we compute the deformation energy (E^{def}) by comparing the total-energy of a isolated molecule constrained to the NHC$^{i\mathrm{Pr}}$/Au(111) configuration with a fully relaxed NHC$^{i\mathrm{Pr}}$ molecule. Here we found that for the atop upright configuration $E^{\mathrm{def}} = 83$ meV, while for the upright adatom configuration $E^{\mathrm{def}} = 67$ meV. However, despite being less stable, in the absence of a surface adatom, the atop tilted and upright configurations may occur, given the formation of these geometries is calculated to be an exothermic process.

2.3 Calculation of charge density

The NHC^{iPr} molecule attaches to the Au(111) surface through a combination of chemical bonding and vdW interactions. In Supplementary Figure 3, we present the self-consistent total charge densities, for an isosurface of $0.02\,e/\text{Å}^3$, of the NHC^{iPr}/Au(111) systems. We can identify the formation of chemical bonds between the NHC^{iPr} molecule and the Au surface atom (adatom) in the atop upright (adatom upright) configuration. Within the same isosurface scale, we find no charge density overlap at the NHC/surface interface for the flat-lying complex geometry.

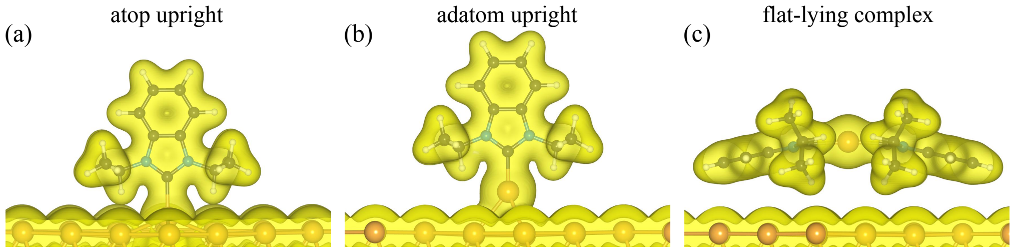

Supplementary Figure 3: NHC^{iPr}/Au charge density (iso surface of 0.02 eV/Å^3) for (a) atop upright, (b) adatom upright, and (c) flat-lying bis-NHC complex configurations.

In order to infer the contribution of the vdW interaction on the NHC/surface binding energies, we remove the vdW contribution from the total energy terms present in E^b, keeping the ground state geometries obtained through GGA+vdW calculations. However, it is worth noting that the GGA approach also brings (although in general underestimated) the energy contributions from long range interactions. We find that the vdW functional contributions on the binding energies of adatom upright and adatom tilted geometries are less then 3.3 %, whereas it increases to 33 % for the flat-lying bis-NHC complex structure. Here, the absence of NHC^{iPr}-Au(111) total charge density overlap (discussed above) and the important contribution from the vdW interaction support the experimentally observed high mobility of NHC^{iPr} complexes on the surface; the complex does not make a strong covalent bond as it does in adatom-attachment.

2.4 X-ray photoelectron spectroscopy

Elemental compositions of samples were evaluated by running widescan at 160 eV pass energy. These samples showed the presence of O, C, and Au. High resolution scans of O 1s, N 1s (except blank), C 1s, and Au 4f were done at 20 eV pass energy. All spectra were charge corrected to Au $4f_{7/2}$. A Shirley background correction was performed on all high-resolution spectra. Au 4f peaks fitted using Doniach-Sunjic (DS) function with FWHM constrained between 0.5 and 1 eV.

Supplementary Table 1: Binding energy shifts (in eV) for various NHC-SAMs on Au(111)

**Element**	**Binding Energy (eV)**			
	**Blank**	**NHC^{iPr}**	**^{Et}NHC^{iPr}**	**NHC^{tBu}**
**Oxygen (O 1s)**	532.35	531.66	532.19<br>530.69	531.22
**Carbon (C 1s)**	288.88<br>286.36<br>284.43	286.46<br>284.85	286.73<br>285.23	286.13<br>284.43
**Nitrogen (N 1s)**	NA	400.94	401.09	400.42
**Gold (Au 4f7/2)**	84	84	84	84

Supplementary Table 2: Atomic percentages obtained from area report in CasaXPS using processed high-resolution scans of various NHCs on Au substrate

**Element**	**Atomic percentage**			
	**Blank**	**NHC^{iPr}**	**^{Et}NHC^{iPr}**	**NHC^{tBu}**
**Oxygen (O 1s)**	8.01	10.80	7.22	3.38
**Carbon (C 1s)**	27.16	46.36	41.83	50.09
**Nitrogen (N 1s)**	NA	5.58	4.79	5.44
**Gold (Au 4f)**	64.83	37.27	46.16	41.09

Supplementary Table 3: Tabulated information showing the C/N ratio and monolayer thickness

	**NHC^{iPr}**	**^{Et}NHC^{iPr}**	**NHC^{tBu}**
**C/N Ratio**	8.31	8.7	9.24
**Thickness (Å)**	9.0 ± 0.8	1.7 ± 1.1	8.5 ± 0.3

Blank Au

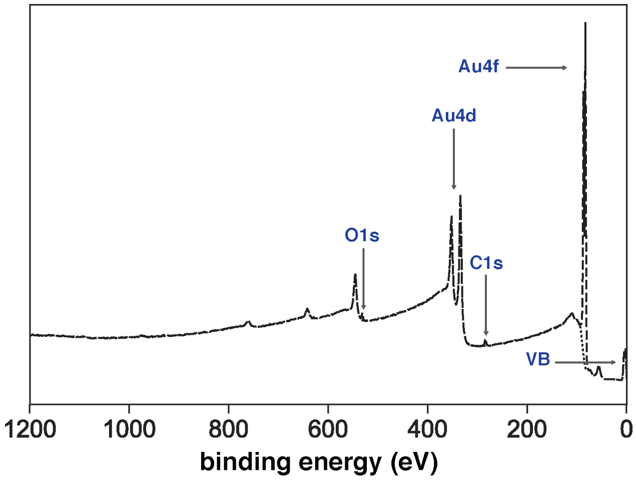

Supplementary Figure 4: Blank-Au widescan XPS showing Au 4f, C 1s, O 1s and VB peaks.

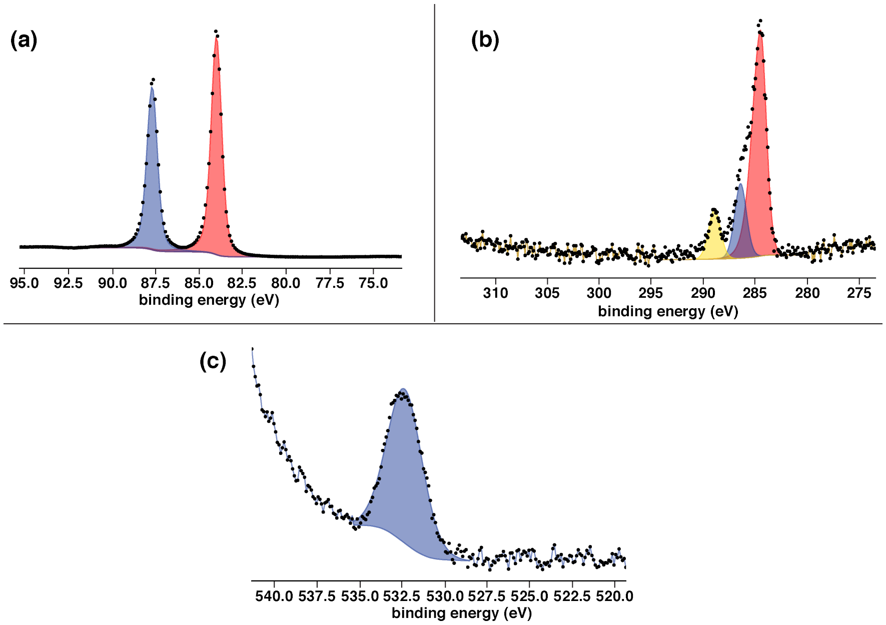

Supplementary Figure 5: Blank-Au high-resolution XPS showing Au 4f (a), C 1s (b) and O1s (c) regions.

C 1s region was fitted to three peaks centered at 284.43 eV (C-C, C=C, and C-H; red peak) and 286.36 eV (C-O; blue) and 288.88 eV (C=O; yellow) using a Voigt function. The FWHM of the peak profile was constrained between 0.5 and 1.5 eV. O 1s region was fitted with one peak centered at 532.35 eV (C-OH and C=O; blue) with a FWHM of 2.45 eV.

NHC^{iPr}/Au

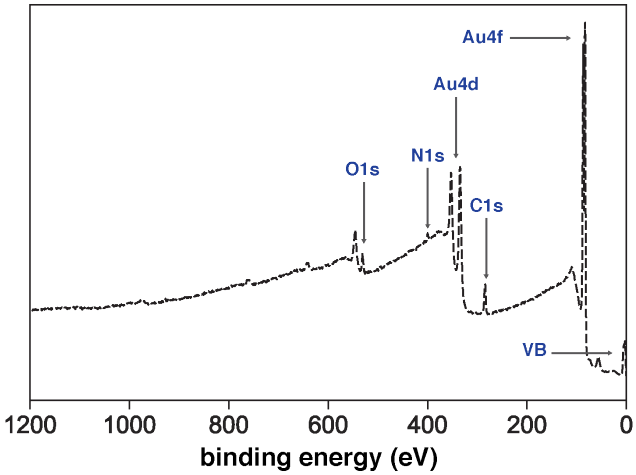

Supplementary Figure 6: NHC^{iPr}/Au widescan XPS showing: Au 4f, C 1s, O 1s, N 1s and VB peaks.

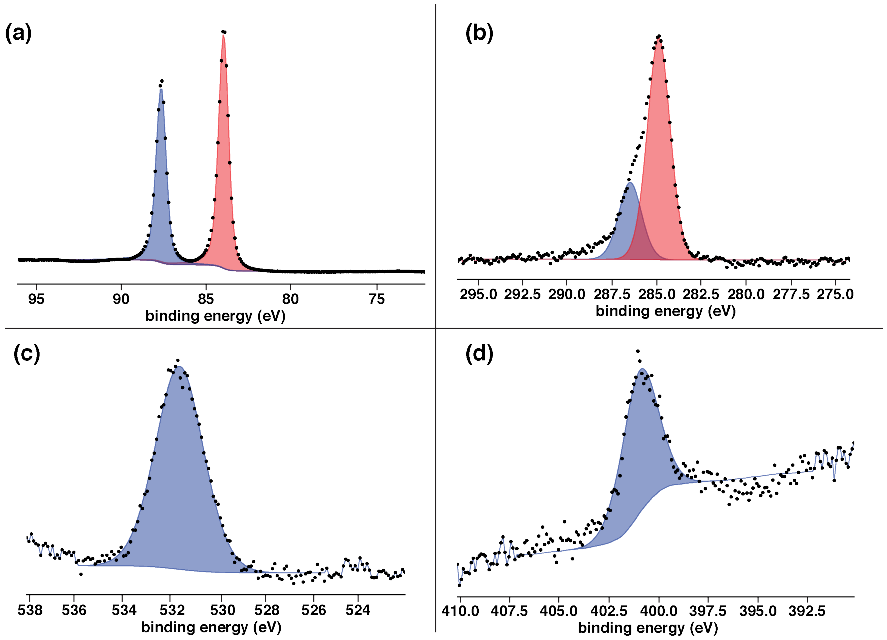

Supplementary Figure 7: NHC^{iPr}/Au high-resolution XPS showing: Au 4f (a), C 1s (b), O 1s (c) and N 1s (d) regions.

C 1s region was fitted to two peaks centered at 284.85 eV (C-C, C=C, and C-H; red) and 286.46 eV (C-N and C-O; blue) using Voigt function. FWHM of the peaks was constrained between 0.5 and 1.5 eV. N 1s region was fitted using a Voigt function with the FWHM constrained between 0.5 and 2.0 eV, and we observed one peak at 400.94 eV indicating formation

of SAM on the Au substrate. The O 1s region was fitted using a Voigt function with one peak centered at 531.66 eV (C-O, C=O (aromatic); blue) with a FWHM of 2.39 eV.

NHC^{tBu}/Au

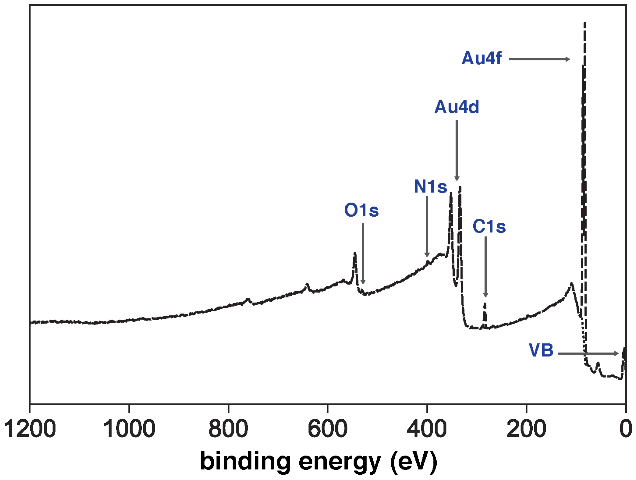

Supplementary Figure 8: NHC^{tBu}/Au widescan XPS showing Au 4f, C 1s, O 1s, N 1s and VB peaks.

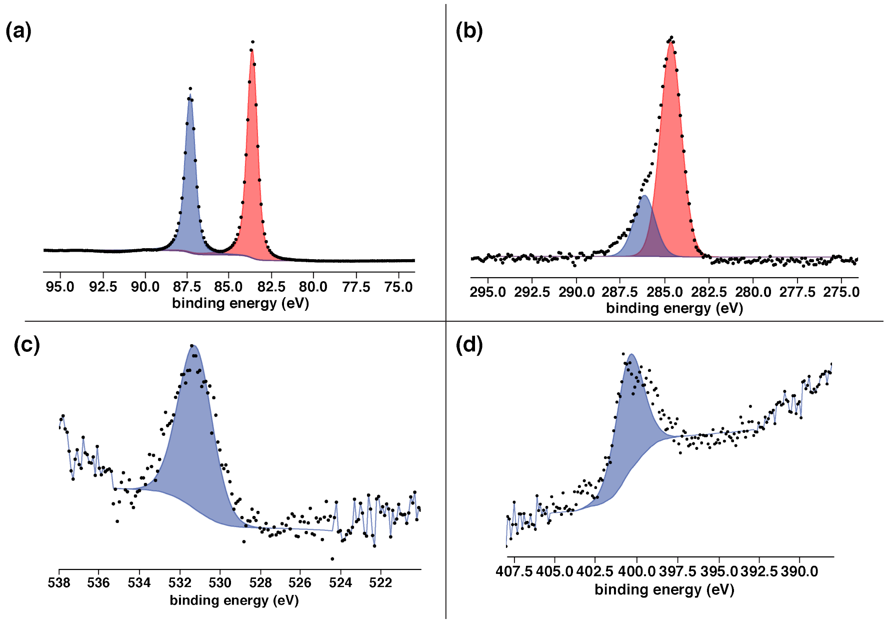

Supplementary Figure 9: NHC^{tBu}/Au high-resolution XPS showing Au 4f (a), C 1s (b), O 1s (c) and N 1s (d) regions.

C 1s region was fitted to two peaks centered at 284.63 eV (C-C, C=C, and C-H; red) and 286.13 eV (C-N and C-O; blue) using Voigt function. FWHM of the peaks was constrained between 0.5 and 1.5 eV. N 1s region was fitted using Voigt function with FWHM constrained between 0.5 and 2.0 eV, and we observed one broad peak centered at 400.42 eV indicating

formation of SAM on the Au substrate. The O 1s region was fitted with a Voigt function with one peak centered at 531.22 eV (C=O (aromatic), N-C=O; blue) with a FWHM of 2.0 eV.

$^{Et}NHC^{iPr}/Au$

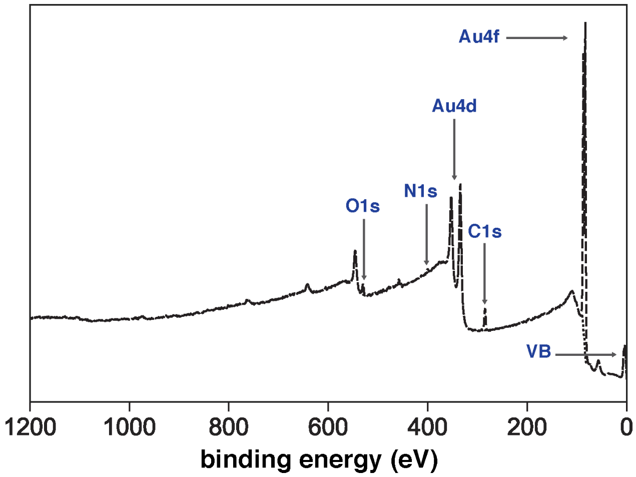

Supplementary Figure 10: $^{Et}NHC^{iPr}/Au$ widescan XPS showing: Au 4f, C 1s, O 1s, N 1s and VB peaks.

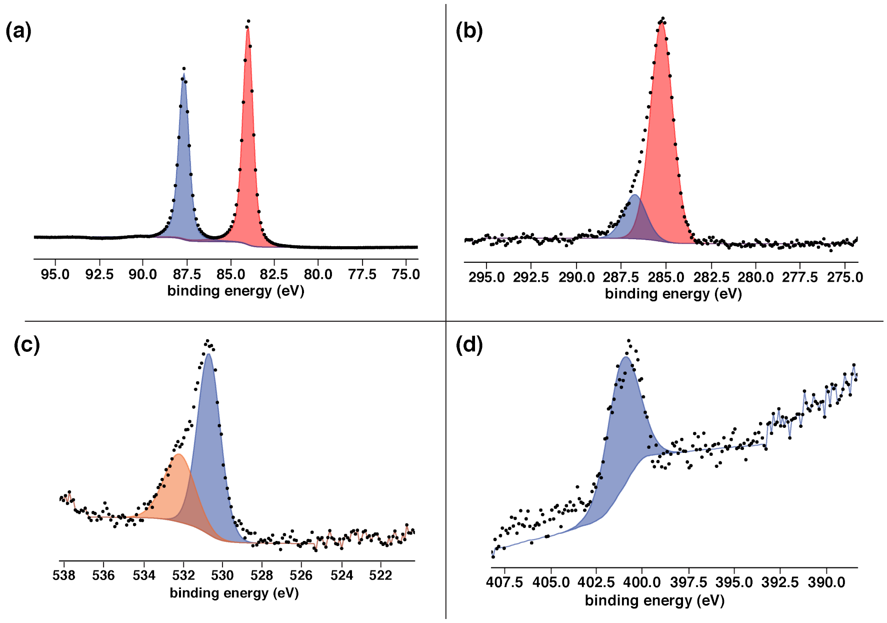

Supplementary Figure 11: $^{Et}NHC^{iPr}/Au$ high-resolution XPS showing: Au 4f (a), C 1s (b), O 1s (c) and N 1s (d) regions.

The C 1s region was fitted with two peaks centered at 285.23 eV (C-C, C=C, and C-H; red) and 286.73 eV (C-N and C-O; blue) using a Voigt function. The FWHM of the peaks was constrained between 0.5 and 1.5 eV. The N 1s region was fitted using a Voigt function with FWHM constrained between 0.5 and 2.0 eV, and we observed one peak at 401.09 eV

indicating the formation of SAM on the Au substrate. The O 1s region was fitted using a Voigt function with two peaks centered at 531.22 eV (C=O (aromatic), N-C=O; blue) and 532.19 eV (C-O-C (aliphatic); red) with a FWHM constrained between 0.3 and 2.0 eV.

2.5 The NHC^{iPr} zig-zag lattice

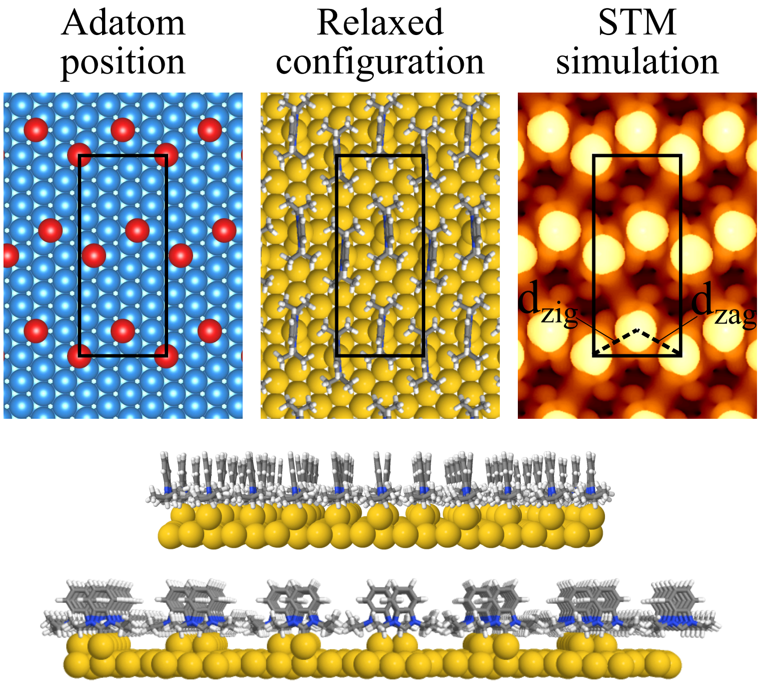

Supplementary Figure 12: Simulation of the experimentally observed zig-zag lattice comprising NHC^{iPr} in the adatom attachment geometry. The STM simulations are in excellent agreement with experiment.

In Supplementary Figure 12, we present DFT simulations of the experimentally observed self-assembled (SA) $(2, 2 \,|\, -8, 8)$ lattice, which is characterized by zig-zag (ZZ) rows of NHC^{iPr}. NHCs in this lattice have a binding energy of $E^b = -2.76\,\mathrm{eV/NHC}^{iPr}$, which is about $0.3\,\mathrm{eV/NHC}^{iPr}$ more stable than the (isolated) adatom NHC^{iPr}/Au(111) configuration, Supplementary Figure 2. These rows of NHCs are nearly 'in-phase', with one row on the hcp hollow sites, and the other on the fcc hollow sites. The structural relaxations were performed by considering the adatom upright geometry as the initial configuration. In this case, due to the symmetry of the substrate, the intermolecular distances, d_{zig} and d_{zag} are the same, 5.87 Å. At the equilibrium geometry (final configuration), the NHC^{iPr} molecules become slightly tilted, and the intermolecular distances are no longer the same. For NHC^{iPr} on the hollow hcp (fcc) sites we found $d_{\mathrm{zig}}/d_{\mathrm{zag}}$ of 5.82/5.76 Å (5.72/5.83 Å). Meanwhile, the C–Au bond lengths (2.56 Å) of the SA molecules are the same compared with that of an iso-

lated adatom upright NHC^{iPr} (Supplementary Figure 2); suggesting that molecule–surface interactions do not change upon self-assembly.

In the same diagram we present the simulated STM image (for unoccupied states) where we found a very good agreement with the experimental image, where our simulated line-profile confirms the experimentally observed lateral distortions resulting in $d_{\mathrm{zig}} \neq d_{\mathrm{zag}}$.

2.6 Height difference calculations

Measurements of height using STM are referred to as apparent height because the tunnel current depends upon tip-sample separation, the local density of states (LDOS) and tunneling matrix elements [3, 4, 5]. The effect of LDOS variations can be minimized using small voltage biases (100 mV rather than 1-2 V) and the effect of the matrix elements can be minimized by probing similar species in similar conformations. A familiar example of this being the measurement of step heights on coinage metal surfaces. In this section STM line-scans, measured with low bias, are presented with calculated line profiles that are based on the experimental parameters. Specifically, we have employed the Tersoff-Hamann approximation [3] to capture the constant current STM, with a charge isosurface of 0.02 $e/\text{Å}^3$ defining the constant current, for the empty states within 100 meV above the Fermi energy. The line-profile Δz is calculated by tracking the height of charge isosurface for those states. In this simulation we have increased the vacuum region to 20 Å in order to avoid spurious periodic charge densities on the images.

Experimental and simulated STM images are shown in Supplementary Figure 13(a-c), which are detailed by line profiles plotted in Supplementary Figure 13(d,e). Supplementary Figure 13(a) shows $\text{NHC}^{i\text{Pr}}$ in a one-dimensional lattice at the step edge and in a zig-zag lattice on the lower terrace. Based on the median height distribution determined by image thresholding, we calculated the apparent height difference for $\text{NHC}^{i\text{Pr}}$ at step edges compared to $\text{NHC}^{i\text{Pr}}$ in the bulk of the zig-zag lattice to be 72±12 pm. The height difference is highlighted by line profiles shown in Supplementary Figure 13(d). Such height value agrees with the *ab initio* line profiles taken from simulated STM images, from which the height difference for $\text{NHC}^{i\text{Pr}}$ on the step edge compared to $\text{NHC}^{i\text{Pr}}$ in the bulk of the zig-zag lattice is 57 pm. For comparison, $\text{NHC}^{i\text{Pr}}$ at the step edge is 60 pm higher compared to the adatom upright configuration, and 130 pm higher compared to the adatom tilted configuration.

DFT calculations show that geometric effects play an important role in the calculated height difference. While molecules in the zig-zag lattice adopt nearly upright configurations (tilted

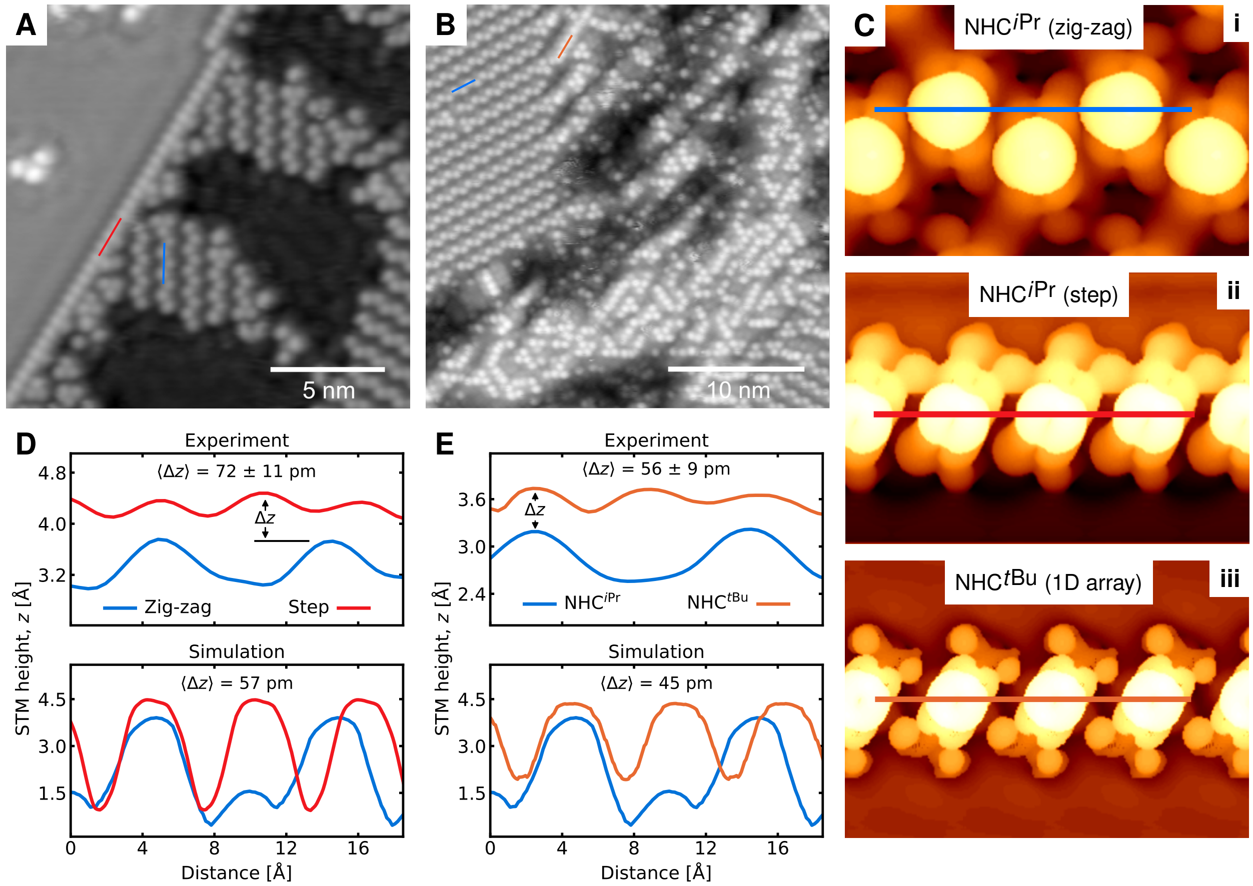

Supplementary Figure 13: (a) NHC^{iPr} molecules decorating the upper Au(111) step edge together with a zig-zag lattice on the lower terrace (20 pA, 100 mV, 18.0 × 18.0 nm^2, 125°C). (b) 50:50 co-deposition of NHC^{iPr} and NHC^{tBu} on Au(111) (20 pA, 600 mV, 30.0× 30.0 nm^2). (c) STM simulations of NHC^{iPr} in the proposed (i) zig-zag lattice and (ii) step edge packing arrangements, and (iii) NHC^{tBu} in the proposed linear arrangement. (d) and (e) are line-scan data providing a comparison of experimental and simulated STM line profiles.

by about 6 degrees), molecules at the step edges tilt towards the lower terrace by approximately 21 degrees. The tilting of NHC^{iPr} bonded to the step edge makes one of its benzene hydrogens higher, by about 30 pm, compared to a non-tilted molecule. Electronic contributions are also found to be significant. For example, the Au–C bond length of NHC^{iPr} on the step edge is 10 pm greater than in the adatom upright/tilted cases.

Supplementary Figure 13(b) shows the results of a 50:50 co-deposition of NHC^{iPr} and NHC^{tBu} on Au(111). NHC^{iPr} and NHC^{tBu} phase-separate, which allows for a direct comparison of their heights. The height difference between linear NHC^{tBu} chains and NHC^{iPr} in the bulk

of the zig-zag array is 56±9 pm. This value agrees with theory, which predicts a height difference of 45 pm (Supplementary Figure 13(e)). This small difference in height suggests that both NHC^{iPr} and NHC^{tBu} attach upright to adatoms.

We find two main contributions to the STM apparent height difference using the equilibrium geometries for the lines and the zig-zag lattice. One comes from the vertical position of the Au adatom attached to the molecule, which is found to be 22 pm higher for the case of NHC^{tBu} in lines, when compared with NHC^{iPr} in the zig-zag lattice. The other contribution comes from the Au–C bond length, 213 and 206 pm for NHC^{tBu} and NHC^{iPr}, respectively. Once the geometrical contributions are accounted for there is a discrepancy of only 16 pm that may be due to the effect of matrix elements and local density of states variations.

2.7 Fine-tuning the intermolecular interactions of NHC^{iPr}

To determine if intermolecular CH-π interactions influence the self-assembly of NHC^{iPr}, we modified the isopropyl wingtip groups with perdeuterated isopropyl groups (NHC^{iPr}-d_{14}). Although this modification should not have a significant impact on steric interactions, it should reduce the ability of the wingtip groups to create CH-π hydrogen bonds [6]. That is, vibrationally averaged CD bonds are approximately 0.5 pm longer than CH bonds, so differences in self-assembly of protonated and deuterated NHCs are unlikely to be driven by changes in steric properties.

In solution, there are several conflicting reports on H/D isotope effects for CH-π interactions, which makes this far from a straightforward comparison. In a report by Shimizu *et al.* [7], it was suggested that H/D isotope effects are minimal or non-existant. However, a different picture emerges from the study of aggregate systems, where small contributions become additive and reveal a likely effect. Consistent with this, studies in condensed systems are more definitive. Isotopic polymorphism is known for 3D crystal structures, with pyridine being one well-known example given by Boese *et al.* [8]. In reviews by Sobczyk *et al.* [9] and Merz *et al.* [10], H/D isotopic effects on hydrogen bonding are observed and attributed to a combination of the lower zero-point energy (ZPE) of deuterium compared to protium, and the anharmonicity of the proton/deutron vibrations. For 3D crystals, intermolecular interactions are often anharmonic resulting in deuterated bridges that are generally weaker than protonated bridges. One important exception is when the potential energy surface is harmonic or close to harmonic, in which case deuterated bridges are stronger. Since a self-assembled monolayer of NHCs can be considered as a 2D crystal, we expect similar rules to apply. For the CH-π interactions examined, the proton/deutron motion is anharmonic, therefore deuterated bridges should be weaker in our assembly.

By comparing Supplementary Figure 14(a) and (b), it is clear that at comparable surface coverages NHC^{iPr}-d_{14} does not form zig-zag arrays like those found on the NHC^{iPr} surface. Both surfaces have a similar concentration of vacancy islands, which suggests that in both

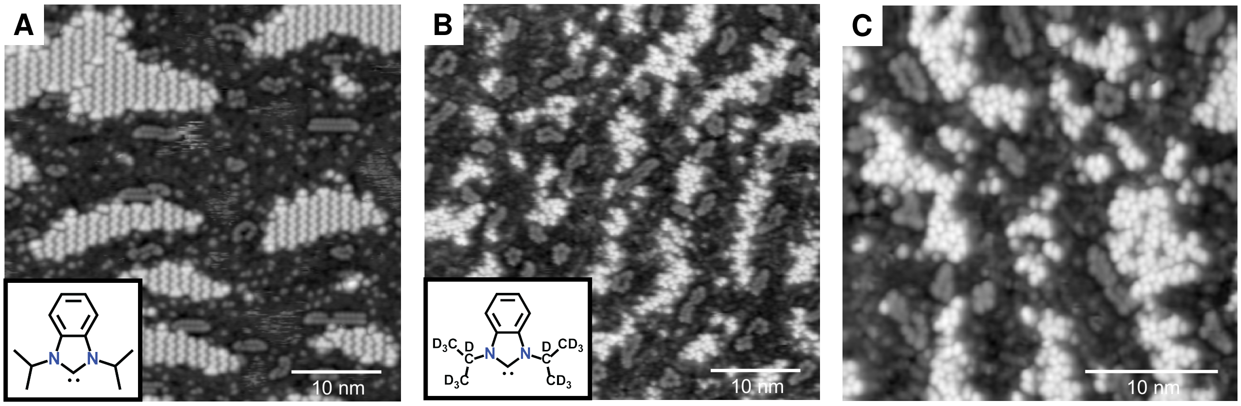

Supplementary Figure 14: The effect of non-deuterated and deuterated isopropyl wingtip groups on the self-assembly of NHC^{iPr} on Au(111). (a) NHC^{iPr} on Au(111) showing the characteristic zig-zag lattice comprised of upright, adatom-bound molecules (20 pA, 100 mV, 45.0× 45.0 nm^2). (b) NHC^{iPr}-d_{14} on Au(111) at a similar surface coverage (30 pA, 800 mV, 45.0× 45.0 nm^2). (c) Detail highlighting its disordered structure (20 pA, 1.0 V, 30.0× 30.0 nm^2).

cases the adsorption process has a similar effect on the gold atoms in the surface layer. An analysis of STM height data in these images suggests that the bright features in Supplementary Figure 14(b) are upright, adatom-bound NHCs. Like NHC^{iPr}, these molecules show a preference for fcc region of the herringbone reconstruction. However, unlike NHC^{iPr}, they do not self-assemble into ordered lattices.

The higher magnification image of the NHC^{iPr}-d_{14} surface in Supplementary Figure 14(c) clearly shows the disordered nature of the NHC^{iPr}-d_{14} overlayer. Since NHC^{iPr}-d_{14} is not observed to self-assemble into an ordered lattice, we conclude that the perdeuterated isopropyl groups do influence the self-assembly of the NHC. Our interpretation of this phenomena is that the attractive lateral interactions are stronger in the case of NHC^{iPr} and this stabilizes the zig-zag lattice. Based on our analysis of the intermolecular separations in the zig-zag lattice, we conclude that the most natural cause of this additional lateral attractive force for NHC^{iPr} is the CH-π interaction.

2.8 Complex self-assembly configurations

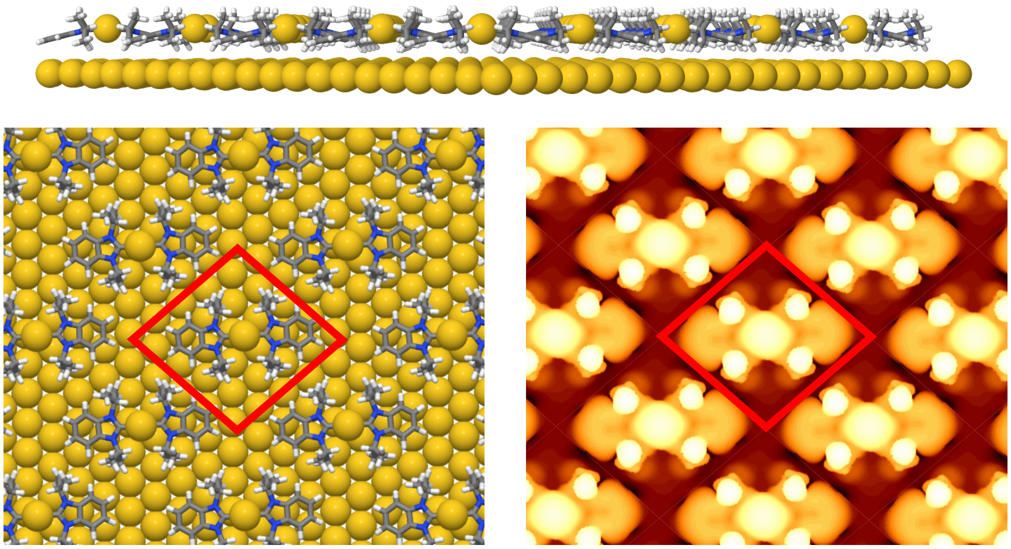

Supplementary Figure 15: Structural model of the $(\mathrm{NHC}^{i\mathrm{Pr}})_2\mathrm{Au}$ complex, forming $(5,-1\,|-1,5)$ lattice on the Au(111) surface, and the simulated STM image of the unoccupied states, corresponding to the experimental parameters.

Supplementary Table 4: Structural parameters (a_1, a_2 and γ) for the complex $\mathrm{NHC}^{i\mathrm{Pr}}$/Au(111) self-assembly supercells, and the binding energy (E^b) for the most stable phase found in each supercell.

System	a_1 (Å)	a_2 (Å)	γ	E^b
$(4,2\,\|-4,6)$	15.5	15.5	81.7^o	-2.542
$(2,4\,\|-5,5)$	15.5	14.7	79.1^o	-2.547
$(5,0\,\|0,5)$	14.7	14.7	60.0^o	-2.578
$(5,-1\,\|-1,5)$	13.5	13.5	82.8^o	-2.583

The calculated binding energies and some key equilibrium geometry information of the simulated complex lattices are summarized in Table 4. It is worth noting that the binding energies of the self-assemblies (i) are very close to that of an isolated $\mathrm{NHC}^{i\mathrm{Pr}}$ complex (E^b=-2.57 eV/$\mathrm{NHC}^{i\mathrm{Pr}}$); and (ii) they differ by up to $\sim$0.04 eV/$\mathrm{NHC}^{i\mathrm{Pr}}$, E^b between -2.54 and -2.58 eV/$\mathrm{NHC}^{i\mathrm{Pr}}$. These findings, (i) and (ii), reveal that, in contrast to the zig-zag lattice, the molecule–molecule interactions play a minor role on the SA; which is consonant with the requirement of spatial constraint in order to form complex lattices. Among the simulated

STM images of the experimental lattice, we found that $(5, -1 \,|\, -1, 5)$ (Supplementary Figure 15, bottom-right) on unreconstructed Au(111) is in best theoretical agreement with the experimental STM result. The $(5, -1 \,|\, -1, 5)$ complex lattice found in Supplementary Figure 15 (bottom-left and top) reveals the formation of a planar sheet of bis-complexes above the Au(111) surface. Unfortunately it is too difficult to calculate possible complex lattice arrangements on the herringbone reconstruction of Au(111).

2.9 Complex lattice instabilities

The zig-zag lattice shown in Supplementary Figure 16 was found to be stable for days and resisted our attempts to manipulate it with the STM tip by changing the set current and voltage and the scan speed and direction. Consequently, we can safely conclude that under normal imaging conditions where we use a low bias voltage (100 mV) and a low tunnel current (20 pA) that the tip does not interfere with the system under study. However, dynamic processes accompany the observation of the flat-lying complex, such as surface diffusion and complex domain rotation, which may be influenced by the presence of the tip and the precise imaging conditions.

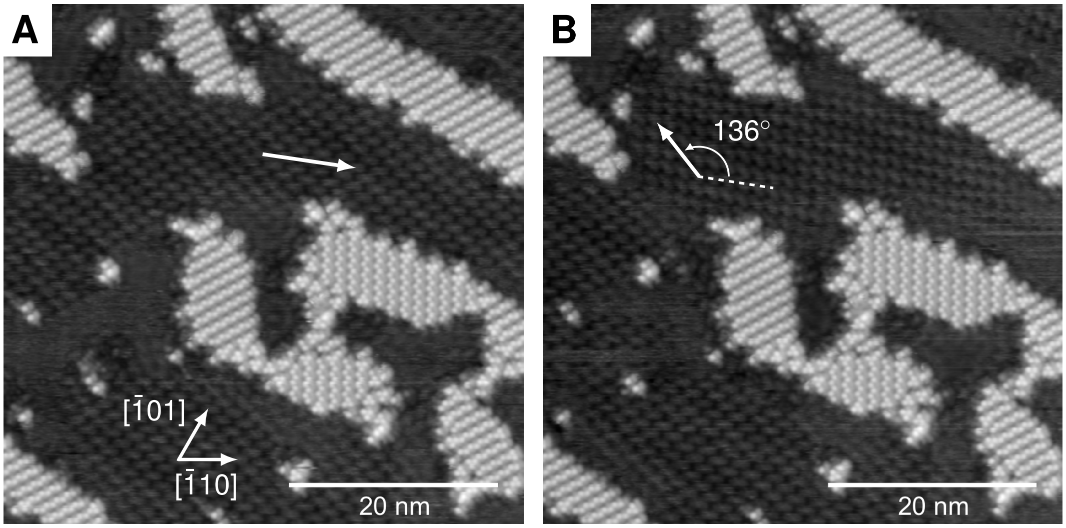

Supplementary Figure 16: (a) and (b) are two successive STM images containing areas with both upright and flat-lying $(NHC)_2Au$ complex lattices (20 pA, 100 mV, 50.0× 50.0 nm^2, 70°C anneal temperature).

The complex lattice possesses a rotational instability that can be seen in Supplementary Figure 16(a) and (b). This surface had been annealed to 70°C to increase the number of complexes and thereby eliminate excluded surface area. In Supplementary Figure 16(a), the white arrow indicates the orientation of the complexes within a domain, which is approximately 8° below $[\bar{1}10]$. The same area was scanned 11.5 mins later and a portion of the domain had rotated by +136°, equivalent to a mirror inversion across $[\bar{1}01]$. These obser-

vations, along with measurements of the lattice vectors, are in agreement with the complex lattice being defined by $(4, 2 \,|\, -3, 6)$. This matrix has equivalent lattice vectors of 1.47 nm when modelled atop the herringbone reconstruction.

Charge density calculations suggest that these complexes do not form a strong covalent bond with the surface. Computational studies suggest these complexes interact with the surface through van der Waals forces, which could explain their high mobility. This is examined in Supplementary Figure 3 where we compare the total charge densities of adatom-bound NHC^{iPr} and complex configurations at the NHC^{iPr}/Au(111) interface.

Repositioning of the wingtip groups for the complexes was also observed. In the STM images, each complex has five maxima (Fig.4(d) in the main text). Four of these are attributed to the isopropyl wingtip groups and the fifth to the gold atom located at the complex center. The conformation of the complexes is seen to switch from a state where the wingtip groups are at the same height above the surface, to a state where two wingtip groups, located diametrically opposite from one another, move away from the surface, resulting in increased brightness for these substituents. Frequently, two or three complexes in a row switch in the same sense. This symmetry breaking conformation change can be followed in sequential STM frames taken typically 2-7 minutes apart at 77 K.

2.10 Diffusion of upright NHC^{iPr} and bis-NHC^{iPr} complexes

Once the zig-zag lattice has formed, we find both upright and flat-lying NHC^{iPr} species on the surface. The surface in Supplementary Figure 17 was prepared from a saturation coverage of NHC^{iPr} on Au(111) at room temperature that was heated to 100°C. In Supplementary Figure 17 we have reproduced four frames from an STM movie; the image details including the time between frames are given in the figure caption. In each frame one can see regions of zig-zag lattice and also, on the left hand side of each frame, NHC^{iPr} attached to a Au(111) step. Also, there are two upright NHC^{iPr} species - a dimer - that are not attached to the zig-zag lattice. These species have the same height as upright NHC^{iPr} in the zig-zag lattice. As a guide-to-the-eye, they have been circled with a white dotted ellipse. In these four frames, the dimer is observed to rotate (frame 1 and 2) and then (frame 3 and 4) take a step closer to the zig-zag lattice.

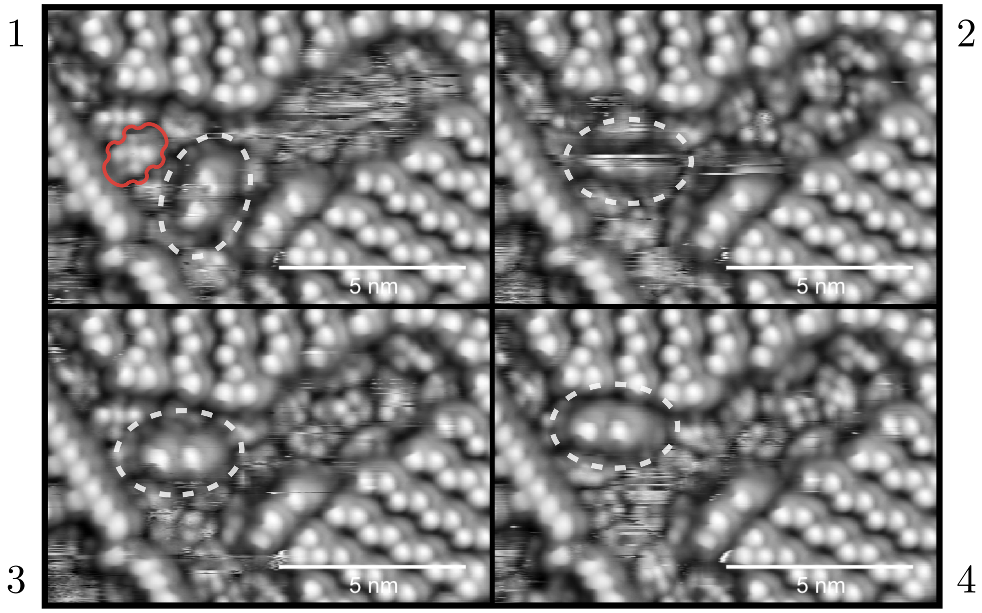

Supplementary Figure 17: Four frames from an STM movie taken at 77 K showing complexes at the edge of a zig-zag lattice (20 pA, 100 mV, 12.0 × 8.0 nm^2, 100°C anneal temperature). The times at the start of image acquisition are: 1) 0 2) 7, 3) 11, and 4) 22 mins. As a guide-to-the-eye, a red contour has been used to outline one complex located just above the step in the first frame. Other features like this can be seen in the four frames. The contrast has been adjusted to enhance the visibility of the flat-lying complexes.

The other features in this image are $(\mathrm{NHC}^{i\mathrm{Pr}})_2\mathrm{Au}^{\mathrm{ad}}$ complexes and one is outlined in red. The complex has one central bright lobe, that our STM simulations associate with the Au complex atom, surrounded by four bright lobes produced by the isopropyl wingtips. In the STM movie, the complexes are observed to move and in some cases the STM has captured them in motion. For example, in the upper right corner of frame 1, streaks can be seen in the (horizontal) fast-scan direction. The streaked region should be contrasted with the same region in frames 2-4 where the complexes are more clearly resolved due to the fact that they are not moving during scan acquisition. In frame 3 one can see complexes that appear to be blocking the movement of the upright dimer. Once, they have moved out of the way, the upright dimer is able to hop towards the zig-zag lattice.

These frames reveal that, in contrast to the zig-zag array which appears static at 77 K, the complexes are in continual motion. A study of STM movies, of this sort, suggests that surface diffusion and self-assembly involves at least two separate populations. It is still an open question whether these populations can be considered to be non-interacting and, therefore, expected to execute random walks, or interacting [11].

Supplementary references

[1] Crudden, C. M. *et al.* Simple direct formation of self-assembled N-heterocyclic carbene monolayers on gold and their application in biosensing. *Nat. Commun.* **7**, 12654 (2016).

[2] Trujillo, M. J. *et al.* Using SERS to understand the binding of N-heterocyclic carbenes to gold surfaces. *The Journal of Physical Chemistry Letters* **9**, 6779–6785 (2018).

[3] Tersoff, J. & Hamann, D. R. Theory of the scanning tunneling microscope. *Phys. Rev. B* **31**, 805–813 (1985).

[4] Blanco, J. M. *et al.* First-principles simulations of stm images: From tunneling to the contact regime. *Phys. Rev. B* **70**, 085405 (2004).

[5] Jelínek, P., Ondráček, M. & Flores, F. Relation between the chemical force and the tunnelling current in atomic point contacts: a simple model. *Journal of Physics: Condensed Matter* **24**, 084001 (2012).

[6] Bates, F. S., Keith, H. D. & McWhan, D. B. Isotope effect on the melting temperature of nonpolar polymers. *Macromolecules* **20**, 3065–3070 (1987).

[7] Zhao, C. *et al.* Do deuteriums form stronger CH-π interactions? *J. Am. Chem. Soc.* **134**, 14306–14309 (2012).

[8] Crawford, S. *et al.* Isotopic polymorphism in pyridine. *Angew. Chem. Int. Ed.* **48**, 755–757 (2009).

[9] Sobczyk, L., Obrzud, M. & Filarowski, A. H/D isotope effects in hydrogen bonded systems. *Molecules* **18**, 4467–4476 (2013).

[10] Merz, K. & Kupka, A. Deuterium perturbs the molecular arrangement in the solid state. *Cryst. Growth Des.* **15**, 1553–1558 (2015).

[11] Barth, J. V. Transport of adsorbates at metal surfaces: From thermal migration to hot precursors. *Surface Science Reports* **40**, 75–149 (2000).
